# 3D facial phenotyping by biometric sibling matching used in contemporary genomic methodologies

**DOI:** 10.1371/journal.pgen.1009528

**Published:** 2021-05-13

**Authors:** Hanne Hoskens, Dongjing Liu, Sahin Naqvi, Myoung Keun Lee, Ryan J. Eller, Karlijne Indencleef, Julie D. White, Jiarui Li, Maarten H. D. Larmuseau, Greet Hens, Joanna Wysocka, Susan Walsh, Stephen Richmond, Mark D. Shriver, John R. Shaffer, Hilde Peeters, Seth M. Weinberg, Peter Claes

**Affiliations:** 1 Department of Human Genetics, KU Leuven, Leuven, Belgium; 2 Medical Imaging Research Center, UZ Leuven, Leuven, Belgium; 3 Department of Human Genetics, University of Pittsburgh, Pittsburgh, Pennsylvania, United States of America; 4 Department of Chemical and Systems Biology, Stanford University School of Medicine, Stanford, California, United States of America; 5 Department of Genetics, Stanford University School of Medicine, Stanford, California, United States of America; 6 Department of Oral Biology, Center for Craniofacial and Dental Genetics, University of Pittsburgh, Pittsburgh, Pennsylvania, United States of America; 7 Department of Biology, Indiana University Purdue University Indianapolis, Indianapolis, Indiana, United States of America; 8 Department of Electrical Engineering, ESAT/PSI, KU Leuven, Leuven, Belgium; 9 Department of Otorhinolaryngology, KU Leuven, Leuven, Belgium; 10 Department of Anthropology, The Pennsylvania State University, State College, Pennsylvania, United States of America; 11 Department of Biology, Laboratory of Socioecology and Social Evolution, KU Leuven, Leuven, Belgium; 12 Histories vzw, Mechelen, Belgium; 13 Department of Developmental Biology, Stanford University School of Medicine, Stanford, California, United States of America; 14 Howard Hughes Medical Institute, Stanford University School of Medicine, Stanford, California, United States of America; 15 Applied Clinical Research and Public Health, School of Dentistry, Cardiff University, Cardiff, United Kingdom; 16 Department of Anthropology, University of Pittsburgh, Pittsburgh, Pennsylvania, United States of America; 17 Murdoch Children’s Research Institute, Melbourne, Victoria, Australia; Shanghai Institutes for Biological Sciences, CHINA

## Abstract

The analysis of contemporary genomic data typically operates on one-dimensional phenotypic measurements (e.g. standing height). Here we report on a data-driven, family-informed strategy to facial phenotyping that searches for biologically relevant traits and reduces multivariate 3D facial shape variability into amendable univariate measurements, while preserving its structurally complex nature. We performed a biometric identification of siblings in a sample of 424 children, defining 1,048 sib-shared facial traits. Subsequent quantification and analyses in an independent European cohort (n = 8,246) demonstrated significant heritability for a subset of traits (0.17–0.53) and highlighted 218 genome-wide significant loci (38 also study-wide) associated with facial variation shared by siblings. These loci showed preferential enrichment for active chromatin marks in cranial neural crest cells and embryonic craniofacial tissues and several regions harbor putative craniofacial genes, thereby enhancing our knowledge on the genetic architecture of normal-range facial variation.

## Author summary

The human face is a highly variable trait composed of distinct features, each influenced by genetic and environmental factors. The strong genetic component is primarily evidenced by the facial similarity between identical twins and the clear facial resemblances within families. Over the past decade, a powerful methodological toolbox of computational and statistical genetics has been developed to study the genetic architecture of complex traits. However, these methods typically require one-dimensional phenotypic measurements (e.g. width of the nose or spacing between the eyes) that fail to accurately describe 3D facial shape. In this paper, we learn from 3D facial data itself, a series of relevant traits that are guided by the facial similarity observed between sibling pairs. Importantly, while preserving the structural convolution of the face, these traits also fit the requirements as input to the well-established statistical tools. In doing so, we have identified many genetic loci that are associated with a wide range of facial features. Some of these regions contained genes related to embryonic facial development, and craniofacial malformations. An improved understanding of the genetic basis of facial shape can have several important applications, for example in developmental biology, medical genetics and forensic sciences.

## Introduction

Systematic characterization of facial morphology is important in a variety of domains such as anthropology, medicine, and genetics [1,2]. It has the potential to provide insight into human evolutionary processes [3], to facilitate surgical planning and outcome assessment [4,5], and to guide syndrome delineation [6], among others. However, fully capturing complex multipartite traits like human 3D facial shape is not straightforward. Traditionally, this has been done using simple anthropometric measurements (e.g. linear distances, angles, and ratios) or principal components (PCs) that are derived from specific points, called landmarks, on a set of two-dimensional (2D) or three-dimensional (3D) facial images [7–16]. However, simple geometric features such as distances fail to capture the full morphological complexity of human 3D facial shape and a priori selection of traits rarely takes into account biological knowledge. As an alternative, Claes et al. [17] recently proposed an open-ended description of facial variation, thereby avoiding any preselection of individual traits. However, highly multivariate phenotypes do not lend themselves to many of the standard tools available in statistical and quantitative genetics, and the number of follow-up analyses therefore remains limited.

**Fig 1:**
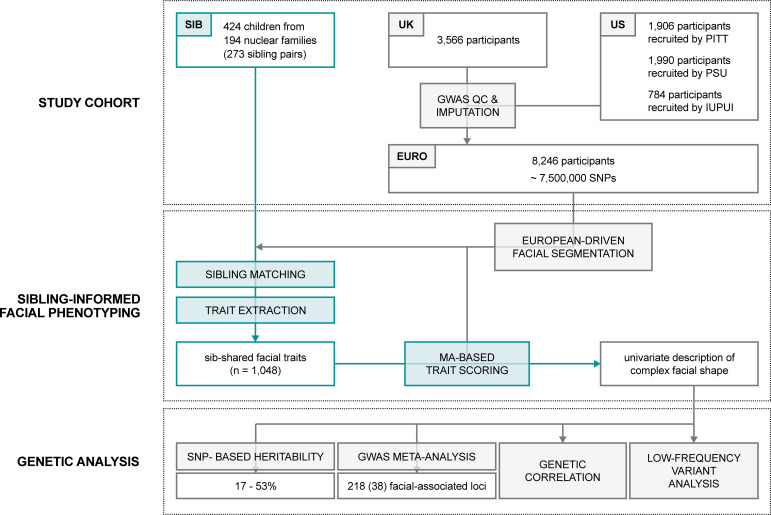
Workflow of the study.

To address the current limitations, we propose to prioritize, in a supervised and data-driven manner, specific facial traits of interest within the multidimensional facial space, in order to reduce the complexity of 3D facial shape into genetically informed and therefore biologically relevant facial traits. In this context, families are potentially informative, as the similarities among family members clearly indicate a heritable component of facial shape [18,19]. Following Fig 1, we aimed to identify facial traits that are shared among sibling pairs in a biometric matching experiment of siblings. Subsequent scoring of a large, separate genotyped cohort for the sib-shared traits provides for the conversion of complex 3D structures into their univariate equivalents so that well-established, open-source bioinformatics tools [20–23] could be applied for further investigation. We sought to identify genetic variants contributing to variation among the sib-shared traits through genome-wide association analysis (GWAS), highlighting a combination of novel and previously identified genetic loci. We further investigated these loci in the context of early craniofacial development and morphogenesis and examined the genetic overlap among sib-shared facial traits. Our analyses have revealed a large number of genetic variants affecting facial traits shared by siblings, illustrating their biological relevance and further enhancing our understanding of the genetic basis of human facial shape.

## Results

### Global-to-local facial segmentation

**Fig 2:**
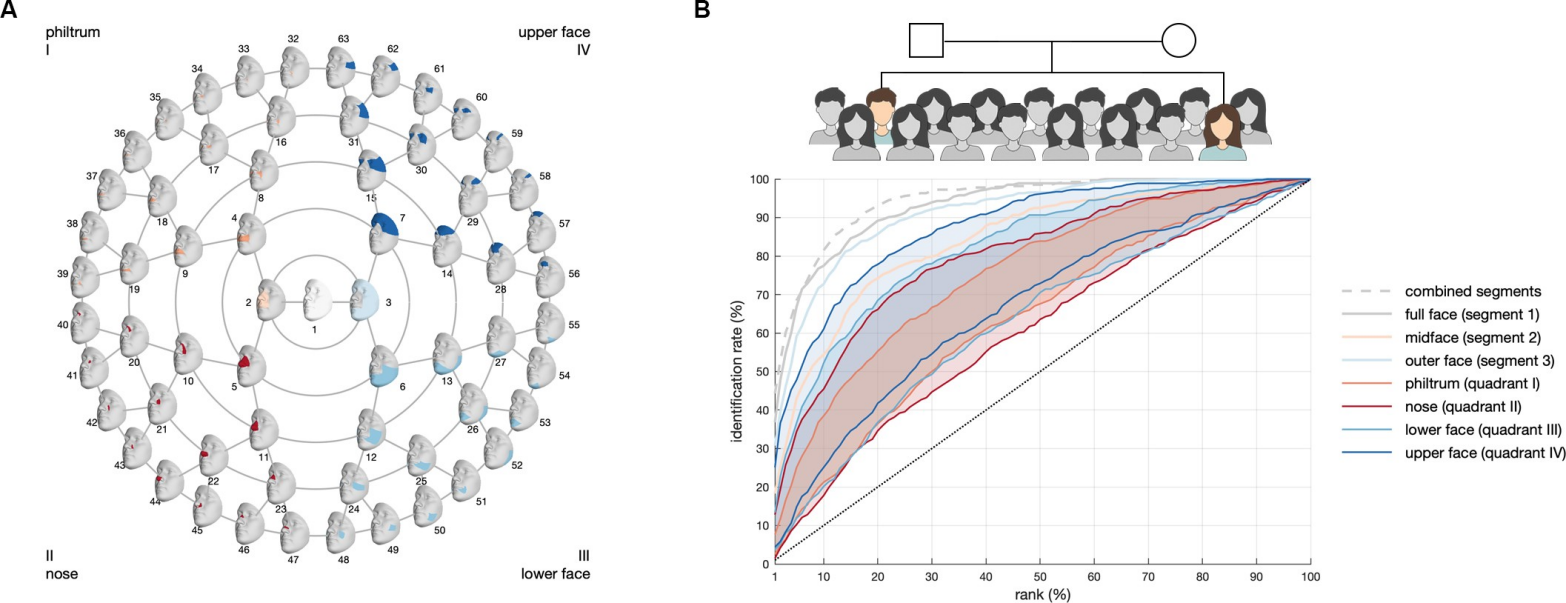
Global-to-local matching of siblings. (A) Global-to-local segmentation of 3D facial shape obtained using hierarchical spectral clustering of the EURO cohort. Segments are colored per quadrant, represented by the roman numerals. (B) Phenotypically similar sibling pairs were identified in a biometric identification setup, which involves the comparison of facial shape between siblings and with unrelated individuals. Matching performance using different similarity measures and facial features was evaluated using cumulative match characteristic (CMC) curves. Plotted is the percentage of sibling pairs that were correctly identified (y-axis) within the top-k% matches (x-axis) using the Mahalanobis angle. Curves are colored based on the facial features that were used to match siblings. For each quadrant, the highest and lowest identification rates per rank are shown, with the area between the two shaded.

Facial variation was studied at multiple levels of scale by subdividing facial shape into 63 hierarchical segments. First, homologous spatially dense quasi-landmark configurations (n = 7,160) were established by the mapping of a generic template mesh onto the images [1,24]. Subsequently, facial shape was hierarchically clustered into a series of global-to-local facial segments in a data-driven manner by grouping quasi-landmarks that are strongly correlated in a large European cohort. We then applied principal component analysis (PCA) to construct a multidimensional shape space for each facial segment independently, the dimensions of which characterize facial variation [17,25]. These segments each captured different aspects of facial shape, representing the full face (segment 1), midface (segment 2) and outer face (segment 3), as well as variations in smaller regions near the philtrum (quadrant I), nose (quadrant II), lower face (quadrant III) and upper face (quadrant IV) (Fig 2A).

### Biometric sibling matching

Since siblings are likely to share facial features due to close kinship, biologically relevant traits can be extracted from phenotypically similar sibling pairs. We aimed to identify these pairs and derive their overlapping facial features through a biometric identification setup, which essentially involves the comparison of facial shape between siblings in the context of multiple facial comparisons with unrelated individuals (Fig 2B). To this end, we used 3D facial images from a total of 273 sibling pairs (78 brother-brother, 79 sister-sister, 116 brother-sister) from 194 nuclear families of self-reported European ancestry (SIB cohort; S1 Table). Individual faces can be described as single points within the multidimensional space constructed per facial segment. Faces that appear to be more similar are closer together within this space, so that distance metrics can be used to measure similarity [26]. Different distance and angle-based measures were quantified, either defined in a Euclidean space (ED, EA) or Mahalanobis space (MD, MA), the latter where each dimension was weighted in terms of its variance [26,27]. In brief, the lower the distance or angle between two points, the greater the similarity between those individuals.

The ability of the different similarity measures to identify siblings was tested in a biometric identification task. In a one-to-many setup, faces were matched against a gallery of candidates for individual and combined facial segments. The rank-k% identification rate then indicates the proportion of times the true sibling was present in the top-k% matches as determined by the similarity score [28]. Therefore, matching at a low rank-k% signifies facially close-to-identical siblings. Overall matching performance of siblings was summarized via cumulative match characteristic (CMC) curves (Figs 2B and S1), where high identification rates and a steep slope of the curve at higher ranks (i.e. low rank values) indicate better performance [29]. To account for the effect of gallery size on the identification performance, results were plotted as a percentage of rank rather than absolute rank values (S2 Fig). In general, angle measurements outperformed distance measurements and performance could be further increased by adjusting for the variances of the PCs in the Mahalanobis space (S1A Fig). Hence, in this study the Mahalanobis angle (MA) was the preferred similarity measure, with the true sibling occurring within the top-1%, top-10% and top-20% candidates in 36.81%, 77.47% and 89.19% of the full-face matching experiments, respectively. While matching at the global, full-face level consistently performed better than individual per-segment matchers (R1_MA,fullface_ = 36.81%; Fig 2B), an increase in performance was observed when all segments branching from the full face (i.e. segments 1–63) were combined (R1_MA,combined_ = 44.32%; S1B Fig). The complete list of matching results for the different similarity measures and segments is provided in S2 Table.

### Data-driven selection of sib-shared traits

Siblings could be matched with varying levels of accuracy depending on the similarity measure and facial features (global-to-local) being tested (Figs 2B and S1). These results suggest that many siblings share one or multiple features in the face, while others look rather different. Derivation of the sib-shared traits was focused on sibling pairs that matched near perfectly (within the top-1%) in any of the segments. We defined the final trait as the average shape of a particular sibling pair within a given segment, highlighting the facial features they have in common, i.e. those that were informative for accurately matching the siblings, while masking their dissimilarities.

**Fig 3:**
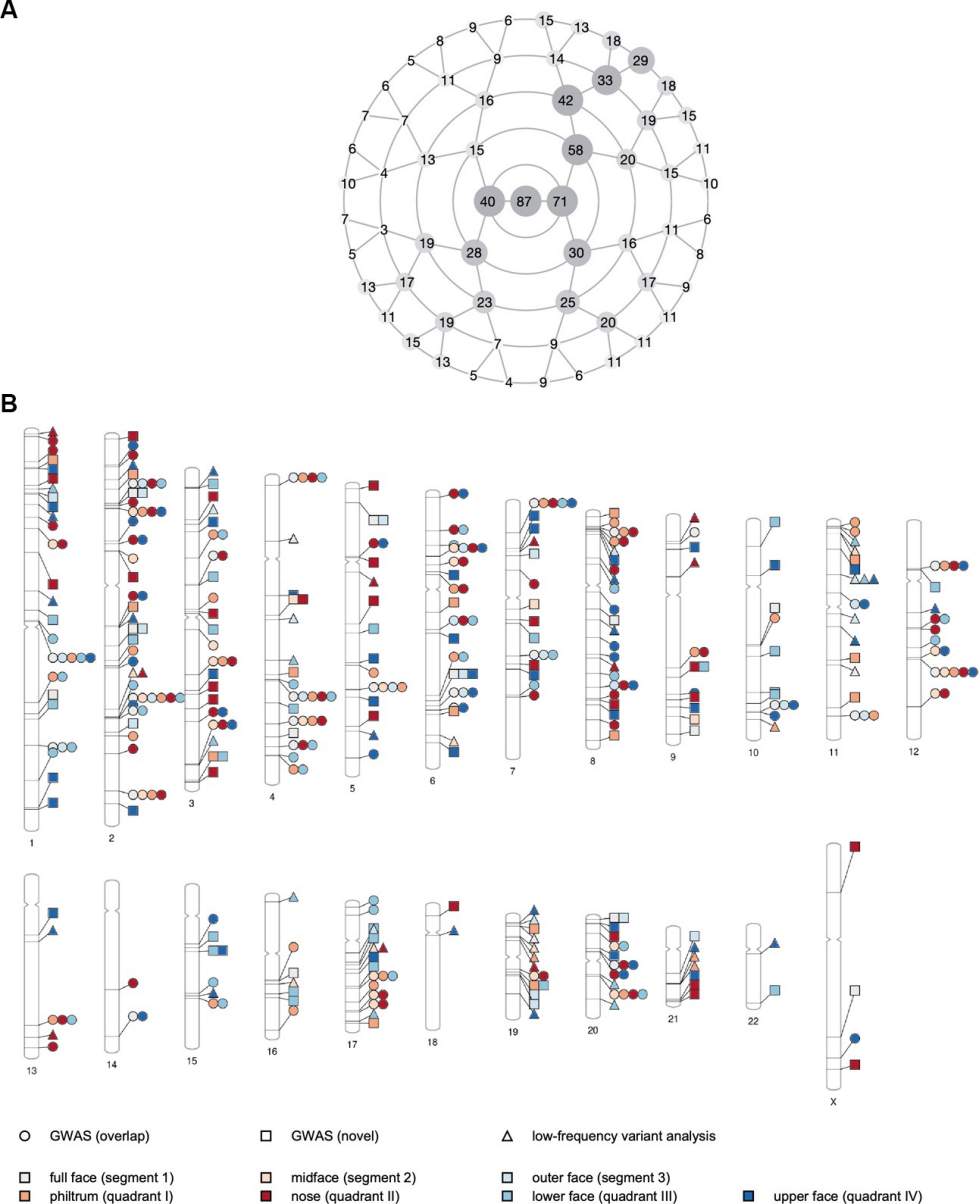
Genetic loci associated with the sib-shared traits. (A) Number of sib-shared traits extracted per facial segment, corresponding to the number of sibling pairs that matched close to perfect within a given segment using the Mahalanobis angle. A total of 1,048 traits were extracted across all 63 segments, comprising 322 independent traits. The structure of the rosette plot corresponds to the polar dendrogram displaying the facial segments in Fig 2A. (B) Ideogram of the genetic loci that contribute to variation in the sib-shared traits, as identified by the association analysis of genome-wide common variants, depicted by circles and squares (i.e. overlapping and novel loci, respectively), and exome-wide low-frequency variants, depicted by triangles. For each locus, the color of the symbol represents the quadrant in which the top associated effect (i.e. lowest p-value) was observed.

A total of 1,048 traits were extracted across all segments, each of them representing a particular facial feature shared by a specific pair of siblings. Visual representations of the sib-shared traits are available online [30]. The 1,048 traits comprised 322 independent traits [31] due to the hierarchical and overlapping construction of the facial segments and the presence of multiple sibling pairs per nuclear family. Facial regions that were more often shared between siblings included the orbital and nasal area as well as the mandible (Fig 3A). From the total of 273 sibling pairs, 218 pairs (60 brother-brother, 66 sister-sister, 92 brother-sister) were used at least once to define the traits, belonging to 160 out of 194 nuclear families. The remaining 55 pairs with poor matching behavior were omitted. No sex effect was observed in the matching of siblings (S3 Fig). That is, relatively equal numbers of same-sex and different-sex pairs were selected (Fisher’s exact test p-value = 0.88) since sexual dimorphism was corrected for during image preprocessing. Similarly, matching of pairs was independent of the age difference of their individuals (two-sample t-test p-value = 0.87).

### Reduction of 3D facial shape to univariate measurements

Sib-shared traits can be described as vectors within the multidimensional shape space, extending from the global mean shape to the average facial shape of both siblings (S4 Fig). Moving further along this direction would produce a more exaggerated version (caricature) of the sibling average, while projecting it in the opposite direction of the global mean generates the inverse or anti-face [32]. In contrast with traditional linear measurements (e.g. distances), each direction or trait may affect multiple parts in the face at once, thus preserving the multivariate and multipartite nature of facial shape. New individuals can also be scored along the sib-shared traits, where the score continuously measures the presence or absence of that particular facial trait in all individuals (S4 Fig). To this end, we used two independently collected samples of unrelated individuals with European ancestry (EURO) originating from the United States (US, n = 4,680) and the United Kingdom (UK, n = 3,566) with genome-wide common variants available (S1 Table). First, SIB and EURO facial images were aligned in dense correspondence, ensuring that homology was established among the 7,160 quasi-landmarks [1,24]. Subsequently, univariate scores were generated by computing the MA between each trait vector and the EURO faces (S4 Fig), since this metric best captured facial trait similarity (S1 Fig). Positive and negative scores corresponded to individuals whose identity vectors [27] were in similar (‘face’) or opposite (‘anti-face’) directions, respectively.

### Genetic determination of sib-shared facial traits

#### Heritability

Narrow-sense heritability of the 1,048 sib-shared traits was estimated from single-nucleotide polymorphisms (SNPs) using GCTA [20,33]. Significance was evaluated according to the false discovery rate (FDR)-adjusted threshold (pFDR_US_ < 3.6 x 10^−3^; pFDR_UK_ < 2.2 x 10^−3^), with significant SNP-based heritability estimates ranging from 0.17 to 0.42 in the US cohort and 0.24 to 0.53 in the UK cohort (S3 Table). Higher values were found on average for traits defined in the global face, nasal area and around the nasolabial folds (S5 Fig). Low SNP-based heritability was observed for traits in small, locally defined areas around the cheeks, philtrum, forehead and chin.

#### Genetic association study

We conducted a genome-wide association scan (GWAS) on all 1,048 sib-shared traits in the US and UK cohorts separately and meta-analyzed the resulting p-values using inverse variance-weighting [22,34] (Figs 3B and S6). We identified a total of 8,944 SNPs at 218 independent loci that reached the threshold for genome-wide significance (p < 5 x 10^−8^), of which 2,749 SNPs at 38 loci had p-values lower than the study-wide threshold adjusted for the effective number of independent tests (p < 1.55 x 10^−10^; S4 Table). The 218 lead SNPs individually explained on average 0.4% and up to 1.6% of the phenotypic variation for individual sib-shared traits. Together, they explained 5.0% to 10.2% for individual traits and approximately 6.6% of the total full-face variation. The LocusZoom plots of the 218 genome-wide significant findings and their associated facial effects are illustrated online [30].

A total of 548 sib-shared traits (52.3%, n = 219 independent traits [31]) were associated with at least one of the 218 genome-wide significant loci, providing 197 traits (18.8%, n = 116 independent traits) that reached the threshold for study-wide significance. Detected associations involved traits in a variety of facial segments, most of them representing variations in the nose (S7 Fig). Several loci showed significant associations with more than one facial region, while others had very localized effects (e.g. the tip of the nose) only [30]. Among the 218 loci, 109 (37 study-wide) overlapped with or were nearby (within 500 kb) the results of prior association studies of normal-range facial phenotypes, providing further support regarding their involvement in facial variation. In addition, we identified 109 loci (1 study-wide) not previously reported in related GWAS literature, some of which harbor putative craniofacial genes as implicated from human malformations (S4 Table). 

In addition to the GWAS meta-analysis, we also studied genetic associations with low-frequency variants (MAF < 0.01) using a gene-based testing approach in a subset of the US cohort (PITT) with exome-wide data available (n = 1,906) (S1 Text). A total of 53 genes passed the exome-wide significance threshold (p < 3.94 x 10^−6^), yet none surpassed the strict study-wide significance level adjusted for the number of independent tests (p < 1.22 x 10^−8^) (Fig 3B and S5 Table). Of these 53 exome-wide significant signals, five could be linked to genes associated with diverse craniofacial phenotypes (S1 Text).

#### Embryonic origin of craniofacial variation

We performed gene ontology analysis using GREAT [35] to study enrichment of biological processes and relevant phenotypes in the vicinity of the 218 genome-wide significant lead SNPs. A significant enrichment was observed for terms related to craniofacial development and morphogenesis (S8 Fig). In addition, our analysis implicated several limb-related processes and phenotypes. Common pathways to both facial and limb development are further evidenced by some ‘cranio-digital’ syndromes [36,37].

**Fig 4:**
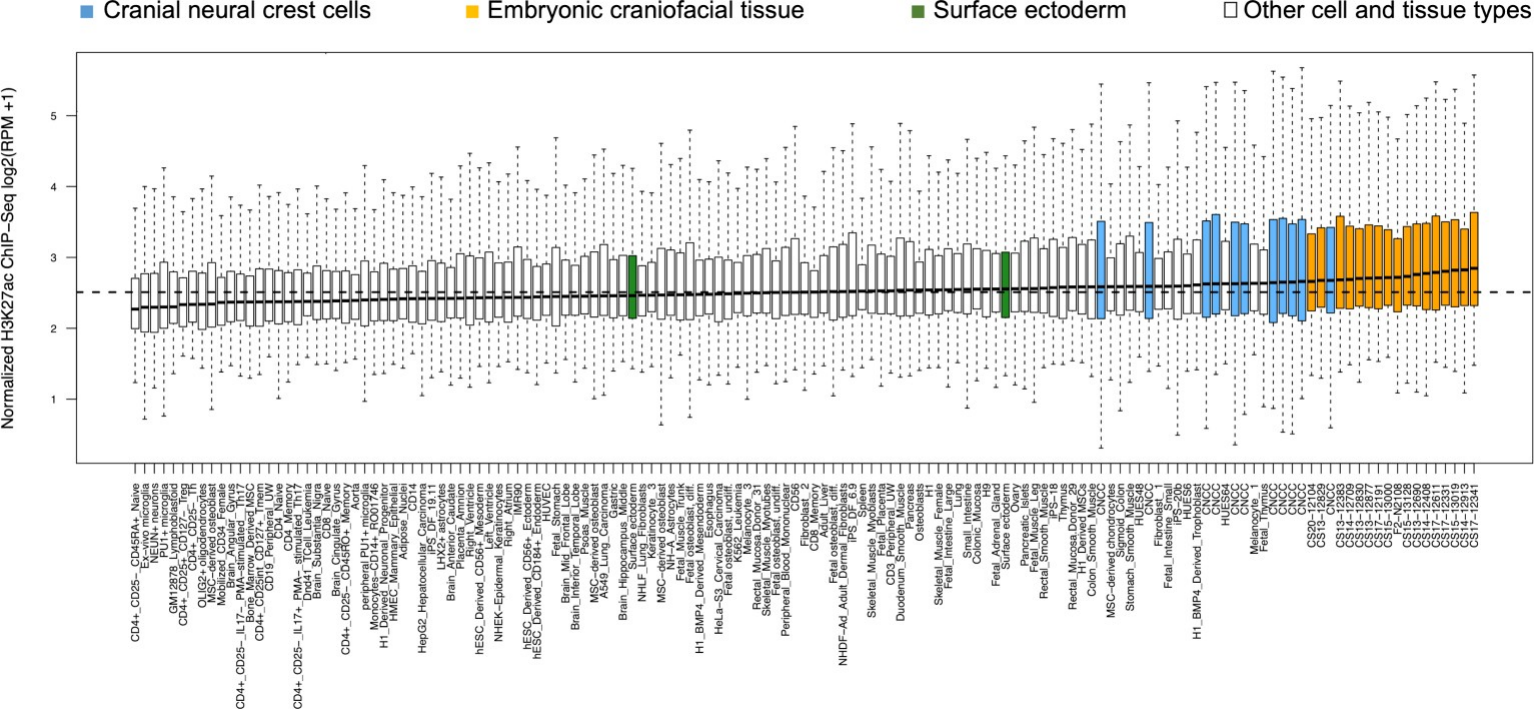
Preferential activity in CNCCs and embryonic craniofacial tissues. Boxplots of the distribution of H3K27ac ChIP-seq signals in 20 kb regions around the 218 genome-wide significant lead SNPs in various adult, embryonic and in vitro–derived cell types. Samples corresponding to CNCCs (blue), embryonic craniofacial tissue (orange) and surface ectoderm (green) are highlighted.

We next sought to identify tissues and cell-types enriched for active regulatory regions near the 218 genome-wide significant lead SNPs. We used ChIP–seq for histone H3 on lysine 27 (H3K27ac), a mark of active regulatory elements, from diverse cell types as described previously [25]. H3K27ac signals near the lead SNPs were most enriched in CNCCs, a transient, embryonic population of cells that give rise to most structures of the craniofacial complex [38,39]. Enrichment was also found for other embryonic craniofacial tissues at different stages of craniofacial development (within first 8 weeks of gestation), suggesting both an early embryonic origin and lasting signals through craniofacial development, respectively (Fig 4).

#### Genetic and phenotypic correlations

We explored both phenotypic and genetic correlations among the sib-shared traits using GCTA (S9 Fig and S6 Table) [20]. High absolute phenotypic and genetic correlations, within and between facial segments, were found together (S9 Fig: diagonal), consistent with the observation that many of the loci identified influence multiple aspects of facial morphology. In addition, environmental factors might also contribute to the observed phenotypic correlations, i.e. for those pairs of traits that had little genetic overlap (S9 Fig: upper left corner).

We also investigated the genetic overlap between our derived facial traits and publicly available non-facial traits and diseases (n = 38; S7 Table) using cross-trait LDSC [21,40] (S2 Text). However, given the relatively modest sample size of the combined EURO cohort (n = 8,246), standard errors for genetic correlations were fairly large (mean = 0.27) and no significant values could be observed after adjustment for multiple testing (p < 4.09 x 10^−6^; S10 Fig and S8 Table).

## Discussion

Over the past decade, a versatile toolbox of computational and statistical genetics methods and accompanying open-access software has been developed to investigate the genetic architecture of complex phenotypes. Examples include, but are not limited to, genome-wide association scans followed by fine mapping [41], rare variant mapping (e.g. burden tests) [23,42], and estimations of SNP-based heritability (e.g. linear mixed models) [20,33] as well as genetic correlations across multiple phenotypes (e.g. LD-score regression) [21,40]. Unfortunately, the deployment of these powerful tools onto the human face is hampered by the multivariate and multipartite nature of facial variability. To overcome this shortcoming, we developed a family-informed strategy for facial phenotyping that reduces 3D facial shape to univariate measurements in a supervised manner, though without resorting to arbitrary and subjective trait selection.

We set up a biometric identification experiment leading to the identification of phenotypically similar sibling pairs from which their overlapping facial traits were derived, each representing global or local aspects of facial morphology that were informative for accurately matching the siblings. Individuals from additional cohorts, independent of their genetic background, can then be scored on the sib-shared traits by measuring the angle between the new individual and the vector of the sib-shared trait, in a common coordinate system. Simultaneously, complex 3D facial structures are converted into univariate descriptors, allowing for the application of contemporary genomic tools. Although traditional univariate, anthropometric measures will remain a valuable resource because of their widespread use and simplicity, they fail to capture the full morphological complexity of 3D facial shape. In contrast, sib-shared traits defined in this work may affect multiple parts in the face at once, thereby preserving its multivariate and multipartite nature. Alternative strategies exist to reduce complex shape to univariate traits such as PCA, where each individual PC constitutes a unique facial trait. However, PCA does not necessarily imply biological relevance, whereas the use of traditional epidemiological approaches (e.g. family-based designs) to drive facial phenotyping facilitates a focus on facial traits that are genetically determined [26,43].

In the biometric identification task, the MA as similarity measure correctly identified the greatest number of siblings from among their peers. Note that, although we use self-reported kinship, the presence of half-sibs or unrelated pairs will have little to no effect as they most likely will not be selected in the matching experiment. Two main observations were made: angular measurements outperformed distance measurements; and Mahalanobis metrics were more discriminative than Euclidean metrics. These results are in line with previous research that demonstrated the role of the angle between vectors, encoded as deviations from a global average, in the perception of facial similarity rather than physical distances [27,32,44]. Moreover, normalization by the standard deviation of each PC in the Mahalanobis space allowed for the reduction of large sources of variation while smaller sources were amplified, so that all types of variation captured by each PC contributed equally when similarity was evaluated [26,27]. O’Toole et al. [45] stated that lower PC dimensions (i.e. those associated with small eigenvalues) convey useful information for distinguishing faces from one another and hence guide recognition. An increased matching performance thus suggests that, besides identity-specific information, family-specific features are encoded in the lower dimensions as well. In view of these results, we used the MA as a metric to score individuals along a given trait direction, thereby neglecting the magnitude of vector. However, the inclusion of the magnitude via distance-based metrics may be valuable beyond the study of normal-range facial variation, for instance within in a clinical context since some dysmorphologies are observed at the extreme ends (distance-based) of the normal spectrum of variation [46,47].

Facial similarity between siblings was studied in the context of different global-to-local facial segments, determined by the structural variations in the EURO reference space. The EURO cohort was chosen as a reference because of its larger sample size in comparison with the SIB cohort, yielding a more robust segmentation of the face of which existing variations are more accurately described following PCA in combination with parallel analysis (S11 Fig). Furthermore, matching of siblings was independent from the selected reference space, either EURO- or SIB-based, when an equal number of dimensions was considered. In contrast, the lower number of PCs originally retained in the SIB reference space following parallel analysis reduced the matching performance, so that the selection of the EURO cohort as a reference is preferred (S11D Fig).

Although matching at the global, full-face level performed better than any individual local matcher, the highest identification rate was achieved through the integration of information across segments. Specifically, almost half of the sibling pairs (44.32%) could be matched within the top-1% when segment-wise MA-corrected features were combined. The improvement of multibiometric systems over unibiometric systems is well-known as they consolidate multiple sources of evidence compensating for the limitations in performance of individual matchers [48]. In other words, siblings will still be matched if they have some, but not all, features in common. This also corresponds to the expected degree of phenotypic resemblance between relatives [49] and highlights the added value of the global-to-local segmentation approach. In this work, we simply concatenated the feature sets of all segments branching from the full face into one single vector, thereby neglecting their correlation structure [50]. However, diverse feature selection methods and fusion techniques exist [48]. In automatic kinship verification literature, accuracies of above 90% are achieved this way [51,52]. In these studies, researchers seek to determine the kin relationship given a pair of 2D images using different feature-based, metric learning and deep learning approaches [51–54]. However, maximizing the performance was not the main focus of this study. Instead, we aimed to identify facial traits that are genetically driven starting from known kin relationships. In this context, we sought to identify obviously matching sib-pairs.

In previous work, we used biometric authentication methods to establish multiple ‘face-to-DNA’ classifiers, each predicting DNA-encoded aspects (e.g. sex, genomic background, individual genetic loci) from facial shape in order to match given faces against a DNA profile [29]. Similarly, we can construct a ‘face-to-face’ classifier as an alternative to the current selection of phenotypically similar siblings based on rank-1% identification rates. For example, a classifier can be trained on the facial similarity between pairs of children for individual and combined segments. When applied to an independent test set of faces, the classifier outputs the probability that the similarity score provided was derived from a pair of siblings. Higher probability signifies greater phenotypic similarity, from which sib-shared traits can be derived. However, classification tasks require the data to be partitioned into proper training and test sets, which reduces the data sample size to work on for specific parts. For example, in such a scenario and in this work only the test data (typically a substantial lower percentage of the total dataset) is eligible for defining sib-shared traits as input to subsequent analyses. Therefore, the number of facial traits as input to the GWAS is very likely to be less, since the likelihood of closely resembling sib-pairs goes down with lower sample size.

Interestingly and affirmative of the proposed methodology, facial regions that were more often shared between siblings (e.g. chin, orbital and nasal region) coincide with regions of high heritability as described independently in previous work [19]. Similarly, average heritability estimated from SNPs was higher for traits defined in the global face and nasal region, consistent with the literature [19,55–57]. Low-to-moderate estimates for traits defined in the regions near the chin and forehead were observed, whereas greater heritability was estimated from twin and family data in these regions using a similar UK cohort [19,58]. This discrepancy could be attributed to the difference in study design (e.g. family or population-based), as SNP-based heritability only provides a lower bound of heritability that is tagged by common SNPs resulting from the genotyping and imputation efforts.

In a genome-wide meta-analysis, we observed 38 study-wide significant loci associated with normal-range facial morphology in individuals of European descent. An additional 180 loci surpassed the conventional genome-wide threshold of significance. Given that association signals close to this threshold are likely to be enriched for genuine signals [59], we report on the full list of 218 loci reaching genome-wide significance. The strongest, study-wide significant signals were found for loci that have been replicated multiple times by independent studies, both within and across different populations. Examples include the well-established genetic regions surrounding *TBX15* [15,17,25,60], *PAX3* [7,8,14,15,17,25,60], *RUNX2* [9,14,17,25,60], *SOX9* [15,17,25,60,61], *PAX1* [9,10,15,25] among others. In addition, promising candidate genes were found near several of the genome-wide significant regions. For instance, the 17q24.1 region was associated with self-reported chin dimpling in the study of Pickrell et al. [60]. This same region showed an association in the current study (p-value = 3.71 x 10^−8^), though the effect was located near the area surrounding the nostrils (trait 339, segment 8). The nearest gene, *AXIN2*, plays a critical role in craniofacial and axial skeleton development of mice [62,63]. The lead SNP rs8080680 is an eQTL of *AXIN2* in blood (GTEx), and overlaps with promoter and enhancer epigenetic marks in many cell types [64]. An association with morphology of the upper lip (trait 763, segment 38) and nasal bridge (trait 376, segment 10) was found in the 9q22.31 region (p-value = 2.39 x 10^−8^). Previous GWAS also identified associations of the same region with aspects of nasal morphology [15,25,60]. The lead SNP rs4275276 is an intron variant of *ROR2*, in which mutations were found to cause a severe skeletal dysplasia known as Robinow syndrome-1 (OMIM 268310). Multiple dysmorphic facial features have been described in patients with Robinow syndrome-1 including frontal bossing, hypertelorism, long philtrum, triangular mouth and a broad nose [65], consistent with the associated effects observed in our GWAS. In a mouse model, *Ror2* has been shown to play a crucial role at multiple sites during organogenesis, providing a developmental basis for the distinct clinical features and anomalies described for Robinow syndrome [66,67].

In addition to the replicated findings, we also identified a total of 109 loci (1 study-wide) that did not overlap with the results of prior GWAS on normal-range facial variation. The 1q25.3 region showed a study-wide significant association (p-value = 6.12 x 10^−11^) with down-turning corners of the mouth (trait 609, segment 25). Among the genes within 500kb of the lead SNP rs6695444, *CACNA1E* is a potentially relevant candidate gene. Mutations in the *CACNA1E* cause developmental and epileptic encephalopathy (DEE; OMIM 618285), a severe and genetically heterogeneous neurodevelopmental disorder characterized by characterized by refractory infantile-onset seizures, severe hypotonia, and profound developmental impairment [68]. Although facial dysmorphism has been described for DEEs caused by mutations in different genes, the link with craniofacial morphology and DEE caused by mutations in *CACNA1E* is less clear. In the study of Helbig et al. [68], macrocephaly was often present in patients with *CACNA1E* mutations, but no other dysmorphic facial features could be observed. Among the novel genome-wide significant loci, some harbor genes known to play a role in craniofacial development or malformations. For example, we observed a signal near *FOXE1* (rs113771540, p-value = 4.29 x 10^−8^) associated with nose (trait 548, segment 21) and chin (trait 625, segments 6) morphology, and a peak spanning *EPHA3* (rs73137393, p-value = 4.49 x 10^−8^) showed an association in the buccal region (trait 422, segment 12). *FOXE1* and *EPHA3* are both orofacial clefting candidate genes [69,70] and have not been associated previously with normal-range facial features. Furthermore, several genes near the GWAS signals have been implicated in human congenital syndromes with craniofacial manifestations. For example, a genome-wide significant association with forehead prominence (trait 447, segment 14) was found in the 20p11.23 region (p-value = 4.78 x 10^−8^). The lead SNP rs6136885 is an intronic variant of the *RIN2* gene. Mutations in this gene were found to cause RIN2 syndrome, formerly known as macrocephaly, alopecia, cutis laxa and scoliosis (MACS) syndrome (OMIM 613075), which is a rare connective tissue disorder characterized by multiple facial and skeletal anomalies [71,72]. Macrocephaly has been described as a clinical feature in some patients, which also corresponds to the facial effect observed in our GWAS. The 8q23.3 region near *RAD21* showed an association (rs4876648, p-value = 3.21 x 10^−8^) with variation in the tip of the nose (trait 224, segment 5). Mutations in *RAD21* result in Cornelia de Lange syndrome (OMIM 614701), a developmental disorder characterized by mild intellectual disability and several facial dysmorphisms [73].

With reference to the study of White et al. [25], in which the same population cohort (EURO) was used, a considerable degree of overlap in the associated genetic loci was observed (n = 100 genome-wide, n = 37 study-wide significant), though both studies also identified a number of distinct regions. The difference between both approaches is that White et al. [25] ran a GWAS on multivariate phenotypes using canonical correlation analysis (CCA). Whereas CCA allows for an open-ended description of facial variation, that is, it extracts the facial effects most correlated with the genotypes, we here searched for specific traits of interest within each facial segment prior to the association scan. Although the percentage of variance explained by the sib-shared traits per segment was generally high (up to 96%), phenotypic variations present in the philtrum and chin regions were described to a lesser extent (S12 Fig). Moreover, decomposition of the H3K27ac signals into those generated by overlapping and novel loci (S13 Fig) demonstrated that enrichment for multipotent, undifferentiated CNCCs, consistent with an earliest embryonic origin, was primarily driven by loci also identified by White et al. [25]. Interestingly, our non-overlapping SNPs showed preferential activity for other relevant embryonic craniofacial tissues that represent progressively later (though still within the first two months of gestation) timepoints in facial development and would thus contain greater amounts of CNCC-derived chondrocytes and osteoblasts, among other cell-types. These findings suggest that the extracted facial traits, which seem to segregate strongly in families, might represent features that form later in craniofacial development. Hence, the use of family-informed strategies alongside an open-ended approach may reveal additional insights into the genetic architecture of complex traits.

When focusing on the genetic correlation among sib-shared traits, we found substantial overlap of genetic variants contributing to variation in these traits. These correlation patterns were in line with the phenotypic correlation, as one could expect from the relationship between both for human traits [74]. Moreover, they also reflect the close embryological relationship of human facial variability. Genetic variation associated with facial morphology might also contribute to various other traits and disease. For example, distinct patterns of facial variation have been described in the literature for various neurodevelopmental and neuropsychiatric disorders with large genetic heterogeneity such as epilepsy [75], autism spectrum disorder [76], schizophrenia [77], and bipolar disorder [78]. Given the univariate nature of the sib-shared facial traits, cross-trait LD score regression (LDSC) can be applied to estimate the genetic overlap with non-facial traits, but much larger sample sizes are required to achieve adequate statistical power [21,40]. Therefore, at the current sample size (n = 8,246), standard errors were large (mean = 0.27) and no significant correlations could be observed (S2 Text).

SNP-based heritability and association signals suggest that common genetic variants contribute to variation in sibling-derived facial traits. As expected, higher SNP-based heritability estimates were found on average for traits that surpassed the threshold for genome-wide significance in our GWAS (two-sample t-test p-value = 4 x 10^−4^ (US) and 5 x 10^−4^ (UK)). However, there still is great overlap in the heritability range between traits that did or did not lead to significant findings when testing for genotype-phenotype associations (S14 Fig and S9 Table). This also limits our ability to further prioritize sib-shared facial traits in terms of their genetic determination in order to reduce the multiple testing burden. The lack of significant GWAS findings for a subset of traits shared by siblings (47.7%) might be attributed to a lack of power to detect variants with weak phenotypic effects, epistatic interactions among variants not picked up by GWAS, and common environmental factors shared by family members. Especially the latter is an important source of bias in family-based designs [49]. In addition, the familial occurrence of particular facial traits such as a square chin [79] suggests that facial features are determined by major gene effects in addition to polygenic effects. Since both common and rare variants can be passed on from parent to child, both can be the reason for siblings to look alike, but the current single SNP-based association approach only supports the identification of common variants. Therefore, we complemented our GWAS with an exome-wide analysis of low-frequency variants, identifying 53 exome-wide significant associations of which eight could be linked to genes associated with diverse craniofacial phenotypes (S1 Text). However, none of the genes tested surpassed the threshold for study-wide significance at the current sample size in the PITT subcohort (n = 1,906; S5 Table).

In conclusion, we describe a data-driven strategy to facial phenotyping, supervised by the phenotypic resemblances between siblings. Traits of interest were then followed up with extensive qualitative analysis using diverse bioinformatics resources. We demonstrate that variability of the sib-shared traits is low to moderately heritable and identify a total of 218 genome-wide (38 study-wide) significant loci associated with normal-range facial morphology. Not only do we provide additional support for numerous previously reported loci, we also identify 109 new genome-wide significant signals (1 study-wide), some of them harboring promising candidate genes as implicated from human craniofacial malformations. Moreover, our analyses indicate a preferential activity of the novel loci in embryonic craniofacial tissue compared with CNCCs, suggesting their action in further differentiated cell-types of the face including osteoblasts and chondrocytes, further complemented by their involvement in limb development as evidenced by gene ontology analysis. A number of follow-up analyses were conducted on the derived set of univariate sib-shared features, such as the analysis of low-frequency variants and genetic correlations using GCTA and LDSC (S1 and S2 Texts). However, only weak associations could be observed, which highlights the need for sample size to increase power. Multivariate methods have been proven to be extremely powerful in dissecting the genetic architecture of craniofacial variation through GWAS for even modest sample sizes [17,25]. Applied to the current sibling-based design, univariate investigations can be followed up with the segment-wise merging and combined analysis of traits derived from a single sib-pair in a multivariate setting. In line with our observation from the biometric matching of siblings using combined segments, we might expect a similar increase in performance and hence statistical power. With regard to potential follow-up studies, a continuing investment in the development of multivariate equivalents to already-existing tools will therefore be of great value to the field. In addition, focusing on specific phenotypes and families, in combination with sequencing technologies, will further enable the identification of rare variants and dominant patterns of inheritance. Such phenotypes can not only be derived from phenotypically similar sibling pairs, as proposed in this study, but extended pedigrees and faces from patients presented with a syndrome might be of great value. Finally, these findings should be followed-up by further replication efforts in larger samples in addition to functional studies in order to elucidate the biological mechanisms that control facial development, with numerous applications in the clinic and beyond.

## Materials and methods

### Ethics statement

Ethical approval was obtained at each recruitment site and all participants gave their written informed consent prior to participation. For individuals under 18 years of age, written consent was obtained from a parent or legal guardian. For the SIB sample, the following ethics approval was obtained: Ethics Committee Research UZ/KU Leuven (S56392: ML10285). For the PITT sample, the following local ethics approvals were obtained: Pittsburgh, PA (PITT IRB #PRO09060553 and #RB0405013); Seattle, WA (Seattle Children’s IRB #12107); Houston, TX (UT Health Committee for the Protection of Human Subjects #HSC-DB-09-0508); and Iowa City, IA (University of Iowa Human Subjects Office IRB #200912764 and #200710721). For the PSU sample, the following local ethics approvals were obtained: Urbana-Champaign, IL (PSU IRB #13103); New York, NY (PSU IRB #45727); Cincinnati, OH (UC IRB #2015–3073); Twinsburg, OH (PSU IRB #2503); State College, PA (PSU IRB #44929 and #4320); Austin, TX (PSU IRB #44929); and San Antonio, TX (PSU IRB #1278). For the IUPUI sample, the following local ethics approval was obtained: Indianapolis, IN and Twinsburg, OH (IUPUI IRB #1409306349). For the UK sample, ethical approval for the study (Project B2261: “Exploring distinctive facial features and their association with known candidate variants”) was obtained from the ALSPAC Ethics and Law Committee and the Local Research Ethics Committees. Informed consent for the use of data collected via questionnaires and clinics was obtained from participants following the recommendations of the ALSPAC Ethics and Law Committee at the time. Consent for biological samples has been collected in accordance with the Human Tissue Act (2004).

### Sample and recruitment

Our study included one family-based (SIB) and one population-based (EURO) cohort. The basic demographic features of both cohorts are provided in S1 Table. For the SIB cohort, data from 647 children from 358 nuclear families were obtained. Families were recruited through various media channels at the Center for Human Genetics (University Hospital of Leuven, Belgium) and at Technopolis, the Flemish Center for Science Communication, Belgium. 3D facial surface scans were available for all children and questionnaires providing information on demographic factors (e.g. sex, age, self-reported ancestry), general physical characteristics (e.g. height, weight), and family relationship were completed by a parent. Only full siblings of self-reported European ancestry, aged 5 to 15 years (median age = 9), were retained for analysis. A further reduction was done by excluding participants with missing data on any of the aforementioned variables and participants with poor quality images. The final study sample consisted of 424 children from 194 nuclear families, containing 273 unique sibling pairs (78 brother-brother, 79 sister-sister, 116 brother-sister; S1 Table). The number of siblings per family ranged from 2 to 5.

The EURO cohort (n = 8,246) included 3D facial images and genotype data of four independent samples, three originating from the US and one from the UK, each composed of unrelated individuals of European ancestry [25] (S1 Table). The US dataset included samples obtained through different studies at the University of Pittsburgh (PITT), Pennsylvania State University (PSU), and Indiana University-Purdue University Indianapolis (IUPUI). Information on demographic factors (e.g. sex, age, self-reported ancestry) and general physical characteristics (e.g. height, weight) were available for all US participants. Individuals were excluded if they reported a personal or family history of any birth defect or syndrome affecting the head or face, a personal history of any significant facial trauma or facial surgery, or any medical condition that might alter the structure of the face. A further reduction was done by excluding participants with missing genotype data, missing covariates, or 3D image artifacts. Lastly, only individuals of European descent were retained, which we identified through projections into a principal component (PC) space constructed using the 1000G Phase 3 dataset (see below). The final PITT sample included 1,906 unrelated individuals (aged 3 to 40 years, median age = 23) from the 3D Facial Norms repository [80]. The final PSU sample consisted of 1,990 unrelated individuals (aged 18 to 88 years, median age = 24). For the IUPUI sample a total of 784 unrelated individuals (aged 7 to 78 years, median age = 19) met all quality-control criteria.

The UK dataset was derived from the Avon Longitudinal Study of Parents and Children (ALSPAC), a UK-based birth cohort study [81,82]. A total of 14,541 pregnant women with an expected delivery date between 1 April 1991 and 31 December 1992, were initially recruited. Extensive information and biological samples have been collected from these mothers and their offspring at various time points, of which details can be found on the study website through a fully searchable data dictionary (http://www.bris.ac.uk/alspac/researchers/data-access/data-dictionary/). Here, 3D facial images, genotype data and self-reported information on age, sex, height, and weight were available for 3,566 unrelated adolescents (aged 14 to 17 years, median age = 15).

### Genotyping, quality control, imputation and population structure

PITT participants were genotyped on the Illumina OmniExpress + Exome v1.2 array, plus 4,322 investigator-chosen SNPs included to capture variation in specific regions of interest based on previous studies of the genetics of facial variation. For the PSU sample, participants were either genotyped on the Illumina Human Hp200c1 BeadChip or on the 23andMe v3 and v4 arrays. Participants from the IUPUI sample were genotyped on the Illumina’s Infinium Multi-Ethnic Global-8 v1 array. Standard data cleaning and quality assurance procedures were performed based on the GRCh37 (hg19) genome assembly using PLINK 1.9 [83]. Specifically, samples were evaluated for concordance of genetic and reported sex, evidence of chromosomal aberrations, genotype call rate (--mind 0.1), and batch effects. SNPs were evaluated for call rate (--geno 0.1), Mendelian errors, deviation from Hardy-Weinberg genotype proportions (--hwe 0.01), and sex differences in allele frequency and heterozygosity.

Genotypes in the PITT, PSU and IUPUI samples, separately, were imputed to the 1000 Genomes Project Phase 3 reference panel [84]. First, pre-phasing of haplotypes was performed in SHAPEIT2 [85], and imputation of nearly 40 million variants was performed using the Sanger Imputation Server [86] with the Positional Burrows-Wheeler Transform (PBWT) pipeline [87]. SNP-level (INFO score < 0.8) and genotype per participant-level (genotype probability < 0.9) filters were used to omit poorly-imputed variants. Finally, a single US cohort (n = 4,680) was obtained by merging the subsamples and filtering the SNPs based on missingness across individuals (--geno 0.5), minor allele frequency (--maf 0.01), and Hardy-Weinberg equilibrium (p < 1 x 10^−6^), ultimately resulting in 7,417,619 SNPs for analysis [25].

European individuals in the US cohort were selected using principal component analysis (PCA) of approximately 450,000 SNPs, after excluding from the imputed data all indels, multi-allelic SNPs, and SNPs with low MAF (≤ 0.1), and SNPs in linkage disequilibrium (50 bp window, 5 bp step size, 0.2 correlation threshold) from the 1000G Phase 3 dataset. A k-nearest neighbor algorithm was used to assign a 1000G population label to each US participant, and those with the 1000G European label of CEU, TSI, FIN, GBR, and IBS were selected for analysis only [25].

For the UK dataset, genotype information was obtained directly from the ALSPAC database. Because restrictions are in place against merging the ALSPAC genotypes with any other genotypes, these were held separately during the analysis. UK participants were genotyped on the Illumina HumanHap550 quad chip platform by Sample Logistics and Genotyping Facilities at the Wellcome Sanger Institute and LabCorp (Laboratory Corporation of America), supported by 23andMe. Genotypes were subjected to standard quality control methods. SNPs were evaluated for minor allele frequency (removed if < 0.01), call rate (removed if < 0.95), and deviation from Hardy-Weinberg genotype proportions (removed if p < 5 x 10^−7^). Individuals were excluded on the basis of gender mismatches, minimal or excessive heterozygosity, disproportionate levels of individual missingness (removed if > 3%), and insufficient sample replication (removed if IBD < 0.8). Only individuals of European descent, compared to the HapMap II dataset by way of multidimensional scaling analysis, were kept for imputation. SHAPEIT2 [85] was used for pre-phasing of haplotypes and imputation against the 1000 Genomes Phase 1 reference panel (Version 3) [88] was performed using IMPUTE2 [89]. The final UK sample consisted of 3,566 individuals with 8,629,873 SNPs for analysis [25].

### 3D facial imaging and shape variables

#### 3D facial image acquisition

3D facial surface images were acquired from all participants using two digital stereophotogrammetry systems and one laser scanning system, applying standard facial image acquisition protocols [2]. For the PITT sample, 3D images were obtained with the 3dMDface camera system (3dMD, Atlanta, GA). Image data of the IUPUI sample were acquired using the Vectra H1 (Canfield Scientific) system, and both 3dMDface and Vectra H1 systems were used for the SIB and PSU samples. For the UK dataset, 3D facial images were captured with the Konica Minolta Vivid (VI900) laser scanners (Konica Minolta Sensing Europe Company, Milton Keynes, United Kingdom).

#### Spatially dense facial quasi-landmarking and quality control

Dense surface registration was performed in Matlab 2017b using the MeshMonk toolbox [1]. In essence, standardized spatially dense quasi-landmark configurations were established by non-rigidly mapping a symmetric template composed of thousands of points (n = 7,160) onto the images [24]. All datasets (SIB, US, UK) were processed separately with the same template, thereby creating homology between the three study cohorts.

Imaging and mapping errors, presented as outlier faces, were detected by a two-step quality control. First, deviations of a face from the global, within-cohort, average were converted to z-scores and images with a z-score larger than 2 were manually checked [17]. A second exclusion criteria involved the percentage of correspondence outliers reported by the MeshMonk toolbox [1], arising from the presence of artifacts such as holes in the facial surface. Similarly, images with scores reflecting a large proportion of outliers were manually checked and removed if necessary.

Standardized and quality-controlled images were aligned in dense correspondence (position, orientation, and size) by generalized Procrustes analysis (GPA). This was done for original and reflected configurations combined, with the latter constructed by changing the sign of the x-coordinate [90]. The average of an original and its reflected configuration constitutes the symmetric component of facial variation, while the difference between the two constitutes the asymmetric component of facial variation. Because faces display bilateral symmetry, aspects of symmetry and asymmetry are preferably considered separately when examining facial shape [91]. Although patterns of asymmetry may be informative, in this work we concentrate on the symmetric component only.

#### Global-to-local facial segmentations

A single EURO cohort was obtained by combining and GPA-aligning the US and UK datasets. We used this large cohort [25] as a reference to perform global-to-local segmentations of the face. Because all individuals, including the SIB cohort, were processed with the same facial template, SIB participants could be projected into the corresponding EURO shape space.

For the three datasets (SIB, US, UK) separately, symmetrized facial shapes were first adjusted for the confounders of sex, age, age^2^, height, weight, facial size and camera system [92] in a partial least-squares regression (PLSR, function plsregress from Matlab 2017b). An additional correction for population structure was performed for both US and UK datasets by including the first four genetic PCA axes (i.e. ancestry axes) in the PLSR model. Next, global-to-local segmentations (Fig 2A) of adjusted faces were performed by grouping vertices that are strongly correlated, characterized by Escoufier’s RV coefficient [93], in a hierarchical spectral clustering approach [17]. Per segment, facial shapes were aligned through GPA, followed by PCA combined with parallel analysis to adequately capture significant facial variations with fewer PCs. In this way, a shape space was established for each segment independently while their integration was preserved through the hierarchical construction. The same segmentation was applied to the SIB dataset and participants were brought into a common global-to-local coordinate system through projections of the SIB shapes into the EURO reference space.

### Data-driven selection of facial phenotypes by matching siblings

#### Measures of facial similarity and sibling matching

Individual faces can be described as single points or vectors within a multidimensional space, whose dimensions characterize facial variation [27,32] (S4 Fig). Image similarity between faces within the PCA space was measured in four ways: Euclidean distance (ED), Mahalanobis distance (MD), Euclidean angle (EA), and Mahalanobis angle (MA). The ED is the simple straightforward linear distance between two points, whereas MD is the variation-normalized version of ED [26]. Normalization was achieved by dividing the PC scores of the SIB cohort by the standard deviation of each PC. Angle-based measurements refer to the cosine distance between two points, treated as vectors, and can be computed from the cosine of the included angle between those vectors. In sum, the lower the distance or angle between two points, the greater the similarity between those individuals.

The ability of the similarity measures introduced above to match siblings was tested using a biometric identification setup for individual and combined segments. The multidimensional PC scores constituted the facial features of each participant for each facial segment. Shape information was combined by concatenating the Euclidean or Mahalanobis-corrected feature sets of multiple segments (segments 1–63) into a single feature vector per participant, similar to feature level fusion in multibiometric systems [48], after which the distance and angle were computed.

In a one-to-many setup, the identification task aimed to identify the one true sibling among a gallery of faces. The gallery was built from the combination of the corresponding sibling and all non-relatives present in the SIB cohort. When multiple (>2) siblings per family existed, the matching experiment was repeated for each pair provided that all other relatives were omitted. Per segment, a total of 546 experiments (2 x 273 unique pairs, as both members of a sibling pair were used as the query image in a separate round) were performed with gallery sizes varying from 417 to 423. In essence, similarity between the query image and all possible candidates in the gallery was measured and ranked in decreasing order from the most likely to least likely candidate. A final score was assigned per experiment reflecting the position of the true sibling in the sorted gallery list. The performance was assessed via cumulative match characteristic (CMC) curves, which plots the cumulative rank-k% identification rate for different values of *k*. High identification rates and a steep slope of the curve at higher ranks (i.e. low rank values) indicated better performance [29].

#### Selection of sib-shared traits

Per segment, sibling pairs were selected if they matched close to perfect (within the top-1%) in the identification task using MA, as this measure performed best in the sibling matching, and when they scored within the lowest 2.5 percentile for the measured similarity. The second constraint was imposed to differentiate between the matching on similarity and distinctiveness (e.g. atypical features) [44] as the latter might form discrete clusters in shape space. In addition, matching behavior had to be symmetric. That is, both selection requirements should have been met regardless of which member of the sibling pair was chosen as the query image. In total, 218 sibling pairs met all selection criteria in at least one facial segment. We defined the final phenotype as the average facial shape of a particular sibling pair within a given segment for which good matching behavior was observed, represented by a vector with reference to the overall EURO mean shape. A total of 1,048 traits were defined across all sibling pairs and facial segments, each of them highlighting the facial features, either global or locally defined, that were informative for accurately matching the siblings. The 1,048 sib-shared traits comprised 322 independent traits [31] due to the hierarchical and overlapping construction of the facial segments and the presence of multiple sibling pairs per nuclear family.

### Conversion of sib-shared traits to univariate scores and genetic analyses

#### Conversion of sib-shared traits

Complex shape transformations, encoded by the sib-shared traits, were converted into simple univariate phenotypes so that well-established toolboxes could be used to assess genetic contributions to the traits. This was achieved by scoring unrelated individuals along the vectors that make up the sib-shared traits. Scores were generated by computing the MA between the trait vector and all EURO individuals, as this measure best captured facial similarity in the matching of siblings, and represented the variation of the sib-shared traits in the EURO cohort (S4 Fig). Individuals whose corresponding vectors had a similar orientation (small angle), independent of their magnitude, received scores close to 1 ([0 1] interval). In contrast, negative scores ([–1 0] interval) were assigned to individuals whose vectors were in opposite directions (large angle).

#### Estimation of SNP-based heritability

For the US and UK datasets separately, SNP-based heritability (h^2^_SNP_) of the sib-shared traits was estimated using GREML implemented in the GCTA package [20,94]. We fitted a linear mixed model with two variance components, including the full genetic relationship matrix between individuals estimated from the SNPs, and the residual variance. The proportion of the total variance explained by all SNPs provided a measure of the narrow-sense heritability. We determined the false discovery rate (FDR) p-value threshold at p < 3.6 x 10^−3^ and p < 2.2 x 10^−3^ in the US and UK cohorts, respectively.

#### Genome-wide association meta-analysis

Similar to the adjustment of facial phenotypes, imputed genotypes were corrected for the effects of sex, age, age^2^, height, weight, facial size, camera system [92], and the first four genetic PCA axes using PLSR. We fitted a linear regression model (function regstats from Matlab 2017b) under an additive genetic model to test for associations between each SNP and each of the sibling-derived phenotypes. For SNPs on the X chromosome, males were coded 0/2 to be on the same scale as 0/1/2 females. Analyses were performed separately for the US and UK cohorts and the resulting p-values were combined in a meta-analysis via inverse variance-weighting [22,33].

We used the conventional threshold of p < 5 x 10^−8^ to claim genome-wide statistical significance. Given the expected number of correlated traits due to the hierarchical design of the facial segmentation and the existence of multiple sibling pairs within a single family, the threshold for study-wide significance was determined at p < 1.55 x 10^−10^ (i.e. p < 5 x 10^−8^ divided by 322), corresponding to an adjustment for the number of independent phenotypes estimated from the eigenvalues of the phenotypic correlation matrix [31].

Because of the large number of signals considered, we used the relatively automated peak detection criterion based on genomic position solely. SNPs that reached the genome-wide threshold were grouped using a 1Mb window. For each region, the lead SNP was defined as the SNP with the lowest p-value for any of the derived traits, resulting in a total of 221 peaks. These peaks were followed up with examination of patterns of linkage disequilibrium (LD) [95], leading to the identification of three regions where association signals were spanning a very large region in the genome. These cases were subsequently merged, refining our results to 218 lead SNPs, all below the genome-wide threshold.

Genes 500 kb up- and downstream of the lead SNPs were identified using the Table Browser of the UCSC Genome Browser [96]. We first investigated whether these genes had any craniofacial relevance by searching the PubMed and OMIM [97] repositories and by looking at the overlap with existing facial GWAS literature. Here, overlap was determined by considering the same +/- 500 kb window around each lead SNP (similar to the definition of peaks) and by investigating LD between the identified and previously reported lead SNPs using LDlink [98]. When no biological plausible candidates were identified, we used FUMA [99] to assign the most likely candidate gene(s) using preset parameters. For each of the lead SNPs, biological functions were annotated using GREAT [35].

#### Cell-type-specific enhancer enrichment

Chromatin activity in the vicinity of the lead SNPs was quantified using H3K27ac ChIP-seq signals from approximately 100 different cell and tissue types, including human CNCCs [100], fetal and adult osteoblasts [101–103], mesenchymal stem cell-derived chondrocytes [101], dissected embryonic craniofacial tissues [104], and iPS-derived surface ectoderm [105], as described in detail by White et al. [25]. As part of this study, we added data from in-vitro-derived surface ectoderm [105]. To compare H3K27ac signals between cell-types in an unbiased manner, we divided the genome into 20 kb windows, and calculated H3K27ac reads per million from each aligned read (bam or tagAlign) file in each window using bedtools coverage. After quantile normalization (using the normalize.quantiles function from the preprocessCore package), we selected the windows containing each of the lead SNPs, random SNPs matched for minor allele frequency and distance to the lead SNPs using SNPsnap [106] and Crohn’s disease-associated SNPs from the NCBI-EBI GWAS catalog [107], the latter serving as a positive control.

#### Phenotypic and genetic correlations

We estimated the Pearson’s phenotypic correlation coefficients among the sib-shared traits. To estimate the genetic correlation between sib-shared traits that is tagged by SNPs, we conducted a bivariate GREML analysis [108,109] of corrected phenotypes in the US and UK datasets separately, as implemented in GCTA [20]. We determined the FDR p-value threshold at p < 1.07 x 10^−5^ and p < 1.00 x 10^−5^ in the US and UK cohorts, respectively.

## Supporting information

S1 Fig Biometric identification results. Cumulative match characteristic curves of (A) the full-face (segment 1) and combined segments for the different similarity measures and (B) individual local matchers and combined segments using the Mahalanobis angle. The diagonal line represents random performance. ED, Euclidean distance; EA, Euclidean angle; MD, Mahalanobis distance; MA, Mahalanobis angle.
(TIF)

S2 Fig Biometric identification versus gallery size. Rank-k and rank-k% identification rates for varying gallery sizes based on the full-face matching of siblings using the Mahalanobis angle. The experiment was repeated 1,000 times, with mean identification rates represented by the solid (rank-k) and dashed (rank-k%) lines, and the minimum and maximum performance indicated by the shaded area. For rank-k% matchings, results are plotted for gallery sizes of 100 and above. In case of rank-1% identification, results are valid only for multiples of 100 as ranks cannot have non-integer values, explaining the decreasing/increasing pattern observed.
(TIF)

S3 Fig Biometric identification results for different sex-based groups. Cumulative match characteristic curves of individual local matchers for the three sex-based groups (n = 78 brother-brother, n = 79 sister-sister, n = 116 brother-sister). Facial similarity was determined using the Mahalanobis angle. The diagonal line represents random performance.
(TIF)

S4 Fig Supervised scoring of individuals onto sib-shared traits. (A) Illustration of a multi-dimensional facial space using PCA, and (B) supervised scoring of individuals onto a specific shape direction coding for the sib-shared trait, depicted by the blue nasal shape. Positive scores indicate the presence of facial features similar to those shared by siblings, while negative scores correspond to features opposite to the sibling pair (left y-axis). The score distribution for all EURO participants is plotted on top of the histogram (right y-axis).
(TIF)

S5 Fig Mean heritability of the sib-shared traits per facial segment. Mean phenotypic variance explained by commons SNPs in the US cohort and UK cohort. SNP-based heritability (h^2^_SNP_) of the 1,048 sib-shared traits was estimated using GCTA and average values per segment are plotted on top of each node. The structure of the rosette plot corresponds to the polar dendrogram displaying the facial segments in Fig 2A.
(TIF)

S6 Fig Manhattan plot of genetic variants associated with the sib-shared traits. Combined Manhattan plot of the sib-shared traits, highlighting the novel and overlapping loci in red and blue, respectively. Per SNP, the lowest meta-analysis p-value across all 1,048 traits is plotted. The solid horizontal line represents the genome-wide significance threshold (p < 5 x 10^−8^) and the dashed horizontal line represents the study-wide threshold (p < 1.55 x 10^−10^).
(TIF)

S7 Fig Genome-wide significant associations per facial segment. (A) Number of genome-wide significant loci that showed an association with one (or multiple) of the sib-shared traits, defined within a particular segment. (B) Proportion of sib-shared traits defined per facial segment (%) that showed an association with at least one of the 218 genome-wide significant loci. The structure of the rosette plot corresponds to the polar dendrogram displaying the facial segments in Fig 2A.
(TIF)

S8 Fig GREAT analysis of the 218 genome-wide significant loci. Top 15 gene ontology enrichment of biological process GO terms, human phenotypes and mouse phenotypes. Plotted is the binomial test FDR (blue) and binomial enrichment (orange).
(TIF)

S9 Fig Genetic and phenotypic correlations among sib-shared traits. Relationship between genetic correlation p-values (x-axis) and the phenotypic correlation (y-axis) in the US and UK cohort. Pairwise correlations between traits that were derived from the same family were excluded. R_p_, phenotypic correlation; R_g_, genetic correlation.
(TIF)

S10 Fig Genetic correlations between sib-shared traits and non-facial traits and diseases. Pairwise correlations between facial and non-facial traits that reached nominal significance (p < 0.05), computed using cross-trait LDSC. Facial traits are sorted per quadrant, corresponding to the polar dendrogram displaying the facial segments in Fig 2A.
(TIF)

S11 Fig EURO- and SIB-based reference space. Hierarchical facial segmentation and number of significant principal components determined by parallel analysis in the (A) EURO (n = 8,246) and (B) SIB (n = 424) cohort. (C) Number of significant components retained by parallel analysis in varying, randomly generated subsets of the EURO cohort. (D) Cumulative match characteristic curves for full-face matchings (segment 1) of siblings based on the Mahalanobis angle in a EURO-based (solid line) and SIB-based (dashed line) reference space. In the ‘SIB70’ space (dotted line), the number of dimensions is equal to the original EURO reference space. ED, Euclidean distance; EA, Euclidean angle; MD, Mahalanobis distance; MA, Mahalanobis angle.
(TIF)

S12 Fig Variance explained by the sib-shared traits per facial segment. The amount of variation explained by the sib-shared traits expressed as percentage for each facial segment. The structure of the rosette plot corresponds to the polar dendrogram displaying the facial segments in Fig 2A.
(TIF)

S13 Fig Preferential activity in CNCCs and embryonic craniofacial tissues. Shown are the boxplots of the distribution of H3K27ac ChIP-seq signals in 20 kb regions around the (A) 218 lead SNPs, (B) 100 overlapping SNPs and (C) 118 non-overlapping SNPs in various adult, embryonic and in vitro–derived cell types. Overlap was determined with reference to the study of White et al. [25], who utilized the same European study cohort in a multivariate GWAS. Samples corresponding to CNCCs (blue), embryonic craniofacial tissue (orange) and surface ectoderm (green) are highlighted.
(TIF)

S14 Fig SNP-based heritability and genome-wide associations of the sib-shared facial traits. Link between SNP-based heritability and (A) study-wide (‘SW’) and (B) genome-wide (‘GW’) significance of sib-shared traits in the GWAS meta-analysis. Traits that didn’t reach statistical significance in the GWAS are coded as ‘0’; traits that were associated with at least one of the identified loci are coded as ‘> = 1’. The two-sample t-test p-value is plotted on top of each panel, with significant values indicated in bold.
(TIF)

S1 Table Summary of the study cohort. (XLSX)

S2 Table Biometric identification results for individual and combined segments. R1, rank-1% identification rate; R10, rank-10% identification rate; R20, rank-20% identification rate; ED, Euclidean distance; EA, Euclidean angle; MD, Mahalanobis distance; MA, Mahalanobis angle.
(XLSX)

S3 Table SNP-based heritability of the sib-shared traits. Significant heritability estimates are indicated in bold. h^2^_SNP_, SNP-based heritability; SE, standard error.
(XLSX)

S4 Table Genome-wide association of common variants with sib-shared facial traits in the discovery samples (US, UK) and combined meta-analysis. The column ’Best Trait’ represents the index (1–1,048) of the sib-shared trait in which the lowest meta p-value (’Best P-value’) was found, together with the corresponding segment and quadrant in which the original trait was defined (’Best Segment’ and ’Best Quadrant’). MAF, minor allele frequency; SE, standard error.
(XLSX)

S5 Table Gene-based association of low-frequency variants with sib-shared facial traits in the PITT subsample. NumVar, number of variants tested per gene.
(XLSX)

S6 Table Genetic correlations among the sib-shared traits. Pairwise genetic correlations in the US cohort (lower/left triangle) and UK cohort (upper/right triangle). Standard errors are displayed between brackets.
(XLSX)

S7 Table Overview of publicly available traits and diseases tested for correlations with the sib-shared traits. (XLSX)

S8 Table Genetic correlations between sib-shared traits and non-facial traits and diseases. Rg, genetic correlation; SE, standard error.
(XLSX)

S9 Table SNP-based heritability and genome-wide associations of the sib-shared facial traits. Statistical significance is indicated in bold.
(XLSX)

S1 Text Exome-wide low frequency variant analysis. (PDF)

S2 Text Genetic correlation between sib-shared traits and non-facial traits and diseases. (PDF)
