# Supplementary figures and images for: 3D facial phenotyping by biometric sibling matching used in contemporary genomic methodologies
Source: PLoS Genet. 2021 May 13;17(5):e1009528. doi: 10.1371/journal.pgen.1009528 (PMC8118281; doi:10.1371/journal.pgen.1009528)

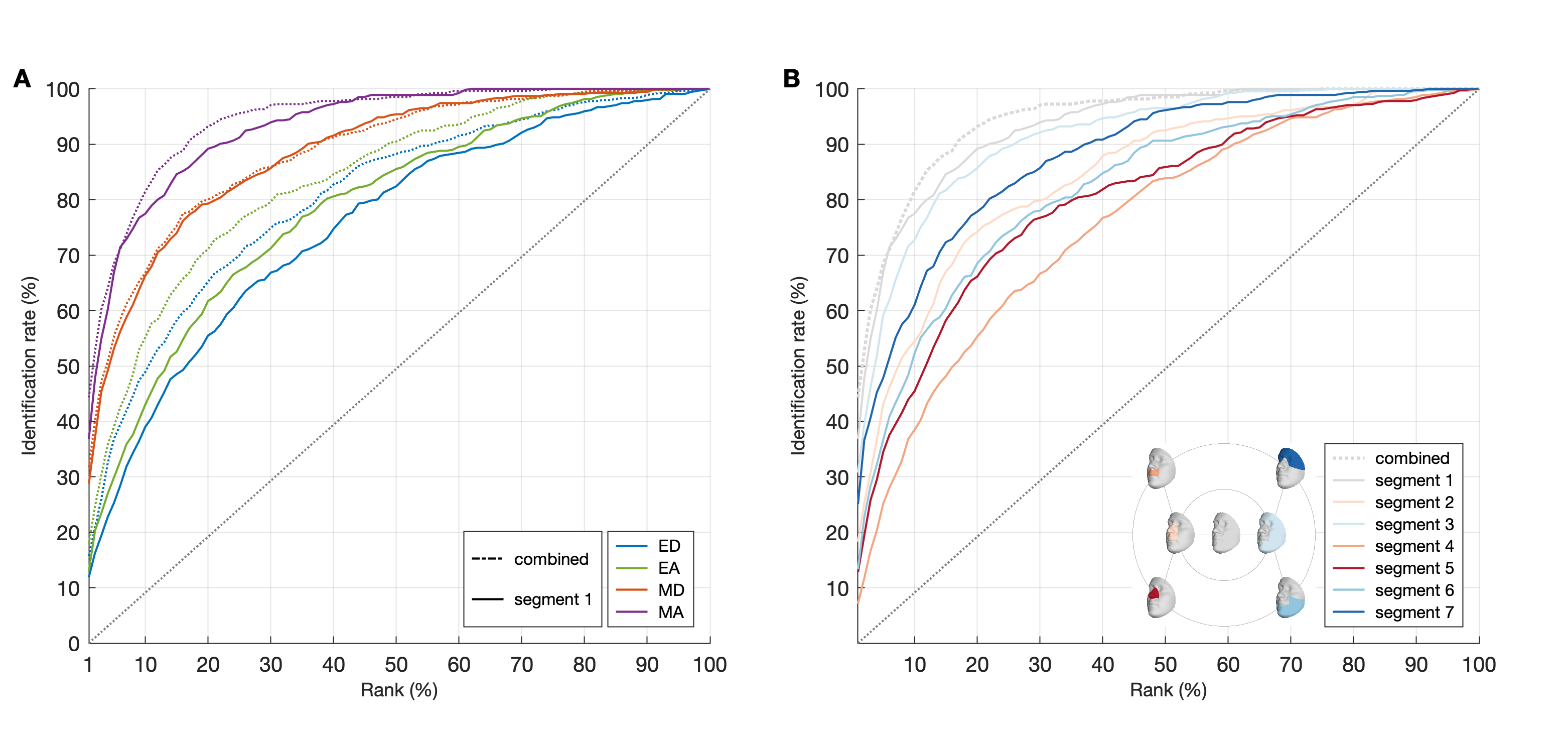

Supplement: S1 Fig — Cumulative match characteristic curves of (A) the full-face (segment 1) and combined segments for the different similarity measures and (B) individual local matchers and combined segments using the Mahalanobis angle. The diagonal line represents random performance. ED, Euclidean distance; EA, Euclidean angle; MD, Mahalanobis distance; MA, Mahalanobis angle. (TIF) [file pgen.1009528.s001.tif]

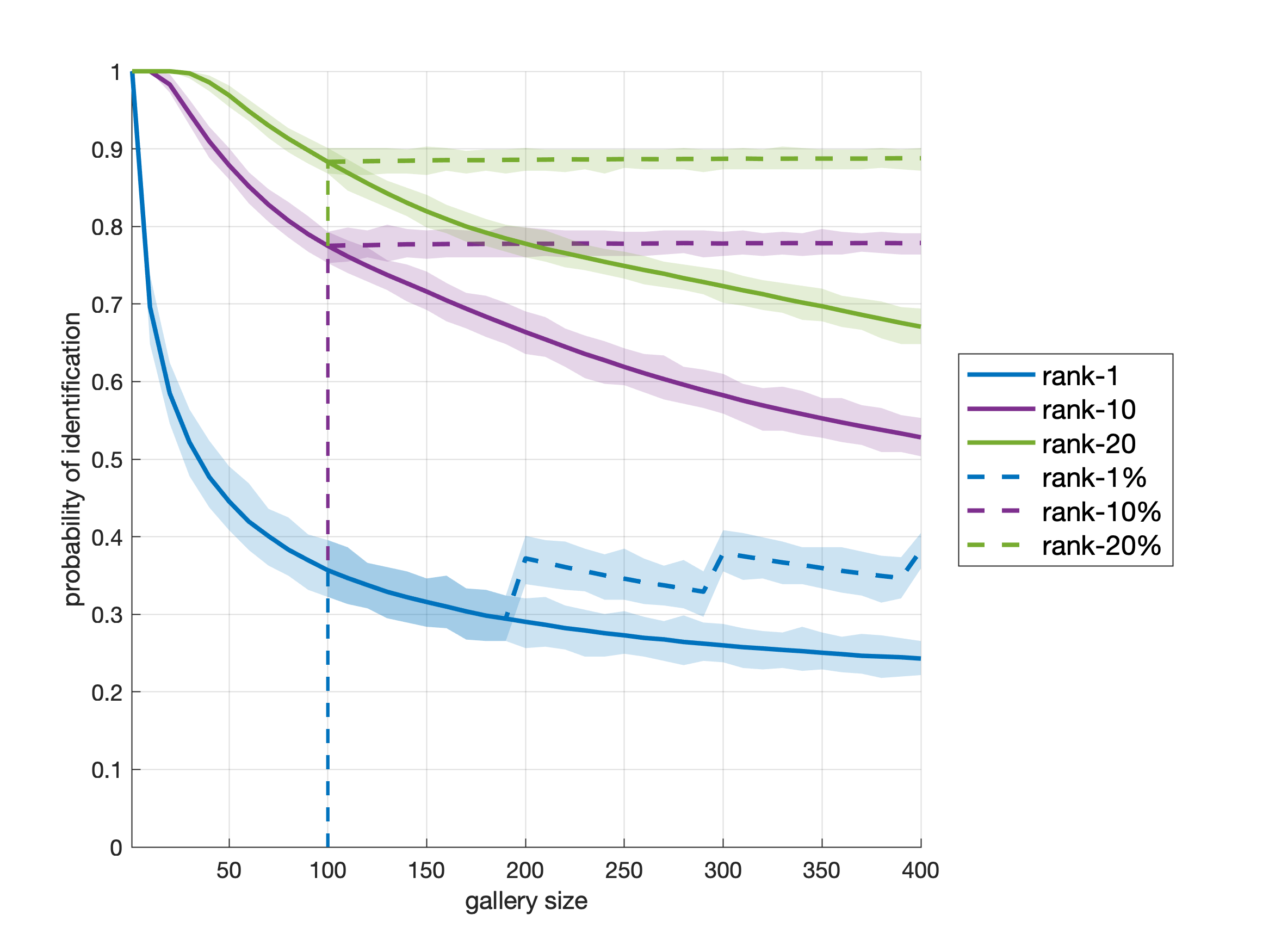

Supplement: S2 Fig — Rank-k and rank-k% identification rates for varying gallery sizes based on the full-face matching of siblings using the Mahalanobis angle. The experiment was repeated 1,000 times, with mean identification rates represented by the solid (rank-k) and dashed (rank-k%) lines, and the minimum and maximum performance indicated by the shaded area. For rank-k% matchings, results are plotted for gallery sizes of 100 and above. In case of rank-1% identification, results are valid only for multiples of 100 as ranks cannot have non-integer values, explaining the decreasing/increasing pattern observed. (TIF) [file pgen.1009528.s002.tif]

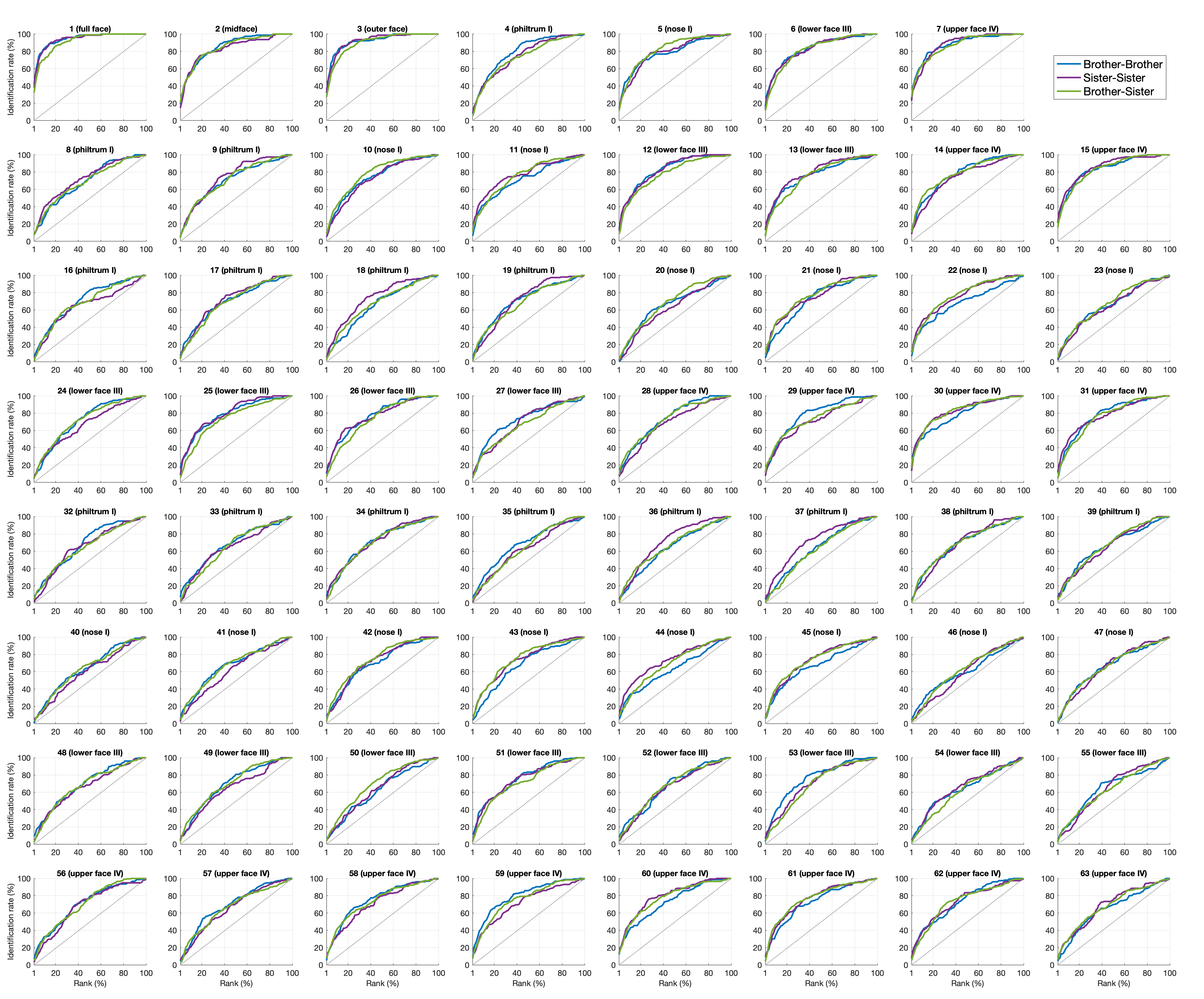

Supplement: S3 Fig — Cumulative match characteristic curves of individual local matchers for the three sex-based groups (n = 78 brother-brother, n = 79 sister-sister, n = 116 brother-sister). Facial similarity was determined using the Mahalanobis angle. The diagonal line represents random performance. (TIF) [file pgen.1009528.s003.tif]

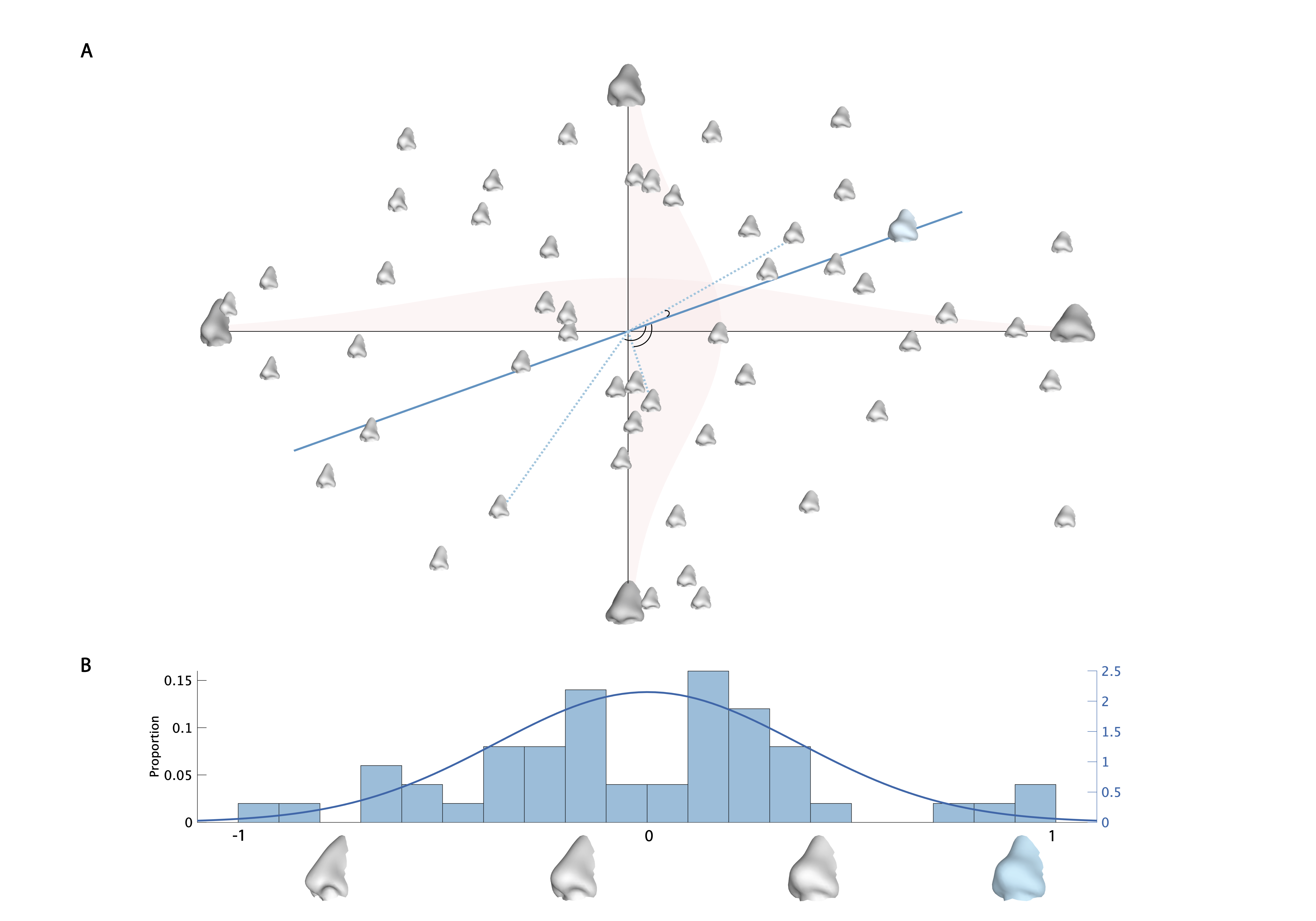

Supplement: S4 Fig — (A) Illustration of a multi-dimensional facial space using PCA, and (B) supervised scoring of individuals onto a specific shape direction coding for the sib-shared trait, depicted by the blue nasal shape. Positive scores indicate the presence of facial features similar to those shared by siblings, while negative scores correspond to features opposite to the sibling pair (left y-axis). The score distribution for all EURO participants is plotted on top of the histogram (right y-axis). (TIF) [file pgen.1009528.s004.tif]

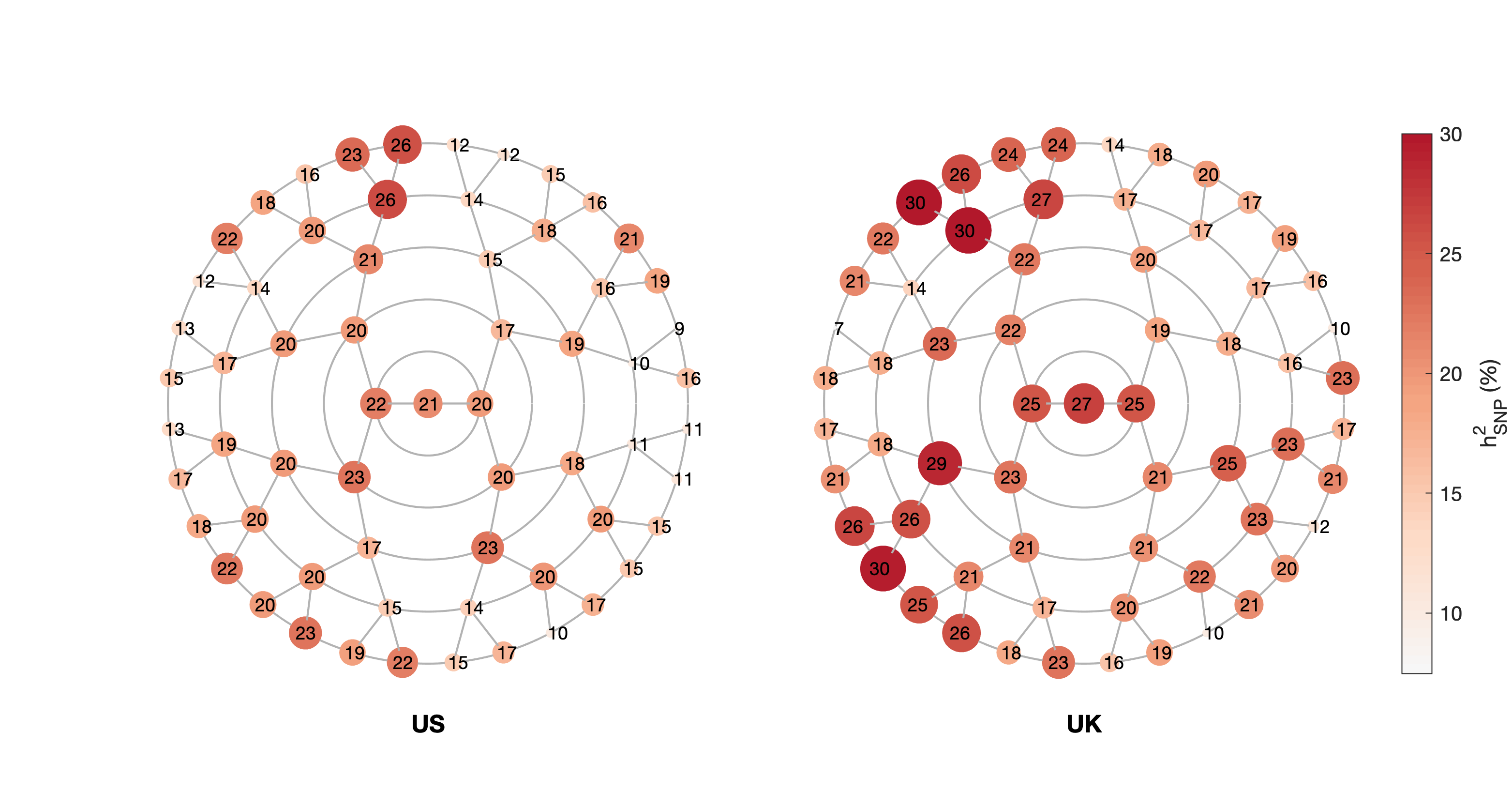

Supplement: S5 Fig — Mean phenotypic variance explained by commons SNPs in the US cohort and UK cohort. SNP-based heritability (h2SNP) of the 1,048 sib-shared traits was estimated using GCTA and average values per segment are plotted on top of each node. The structure of the rosette plot corresponds to the polar dendrogram displaying the facial segments in Fig 2A. (TIF) [file pgen.1009528.s005.tif]

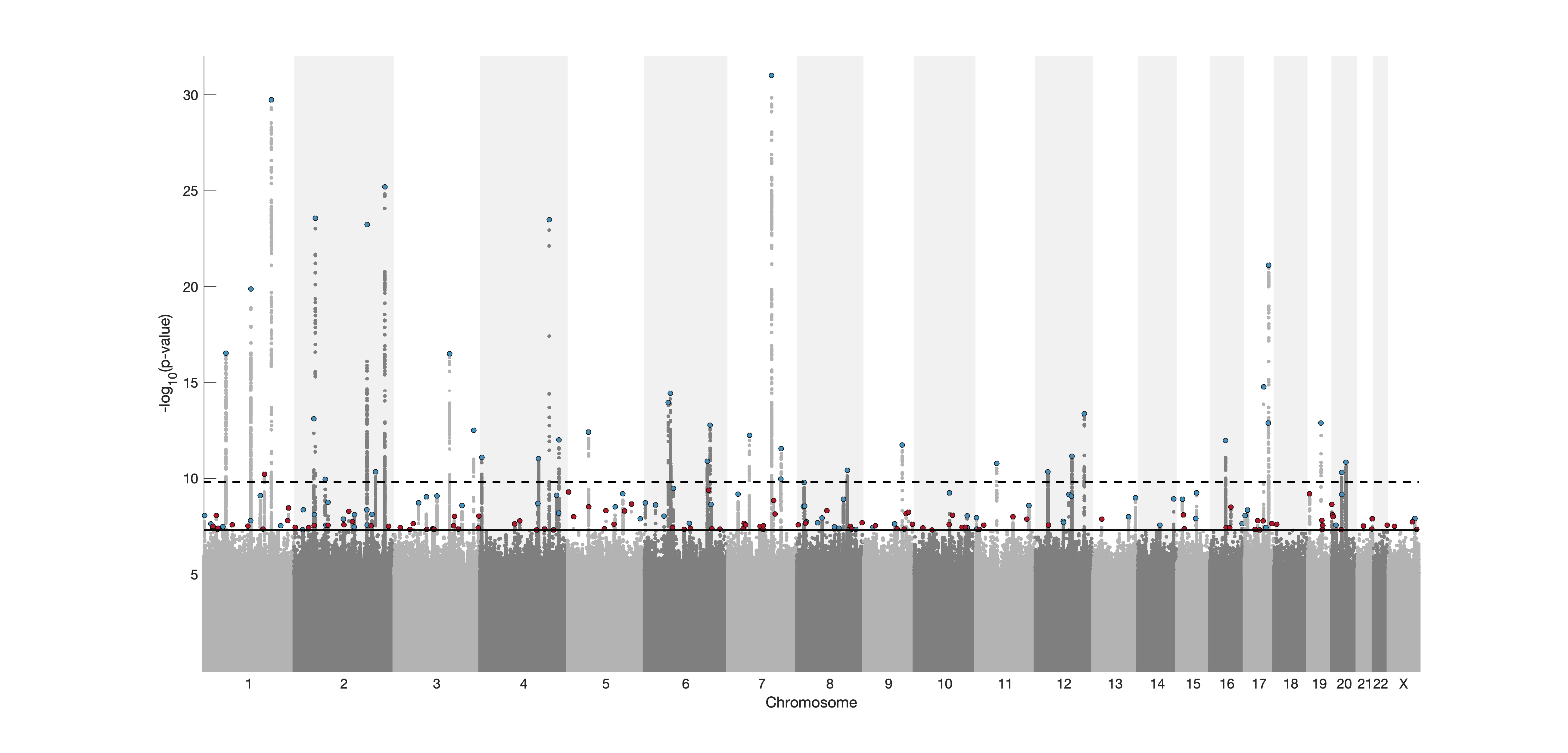

Supplement: S6 Fig — Combined Manhattan plot of the sib-shared traits, highlighting the novel and overlapping loci in red and blue, respectively. Per SNP, the lowest meta-analysis p-value across all 1,048 traits is plotted. The solid horizontal line represents the genome-wide significance threshold (p < 5 x 10^−8^) and the dashed horizontal line represents the study-wide threshold (p < 1.55 x 10^−10^). (TIF) [file pgen.1009528.s006.tif]

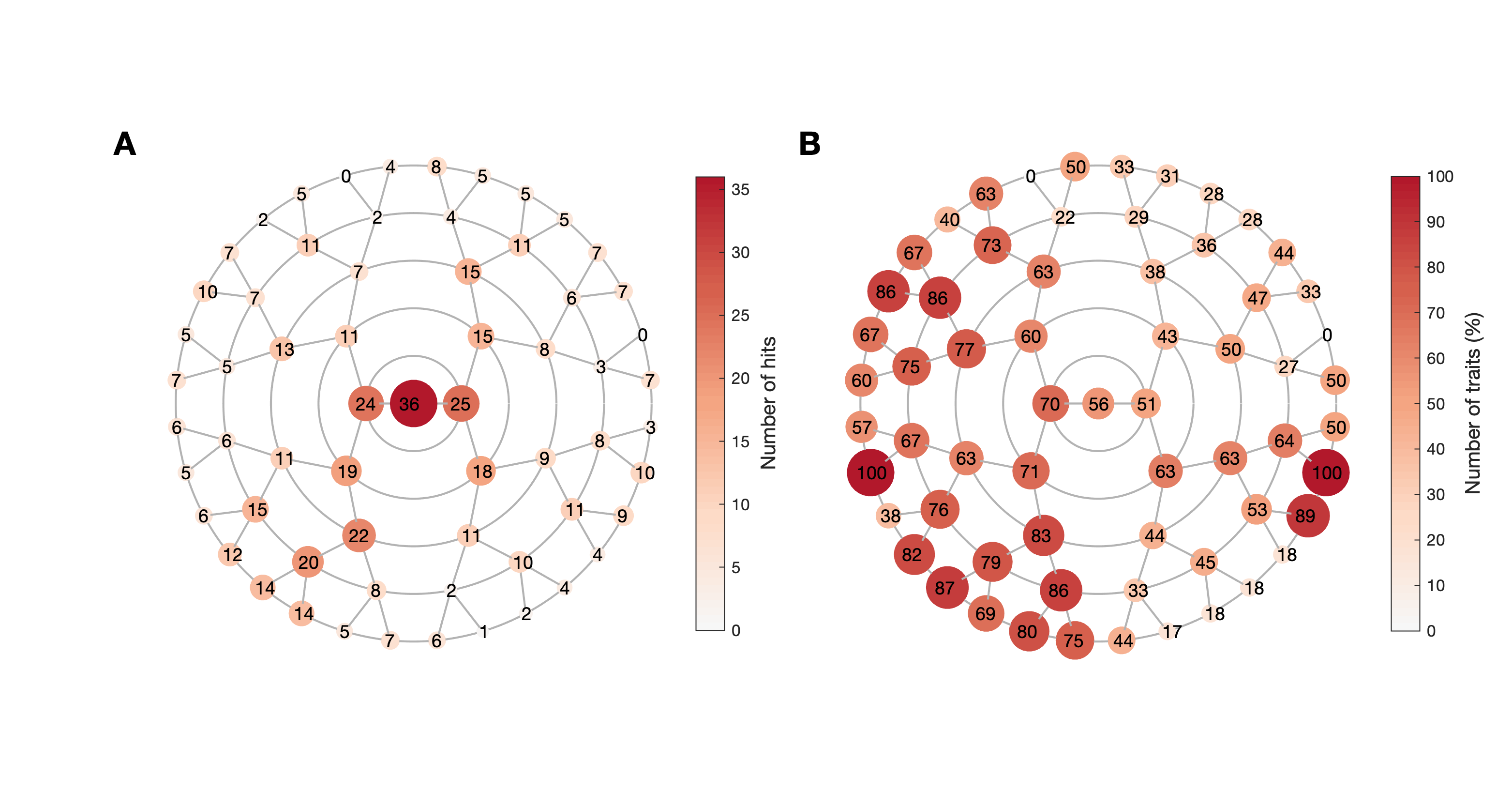

Supplement: S7 Fig — (A) Number of genome-wide significant loci that showed an association with one (or multiple) of the sib-shared traits, defined within a particular segment. (B) Proportion of sib-shared traits defined per facial segment (%) that showed an association with at least one of the 218 genome-wide significant loci. The structure of the rosette plot corresponds to the polar dendrogram displaying the facial segments in Fig 2A. (TIF) [file pgen.1009528.s007.tif]

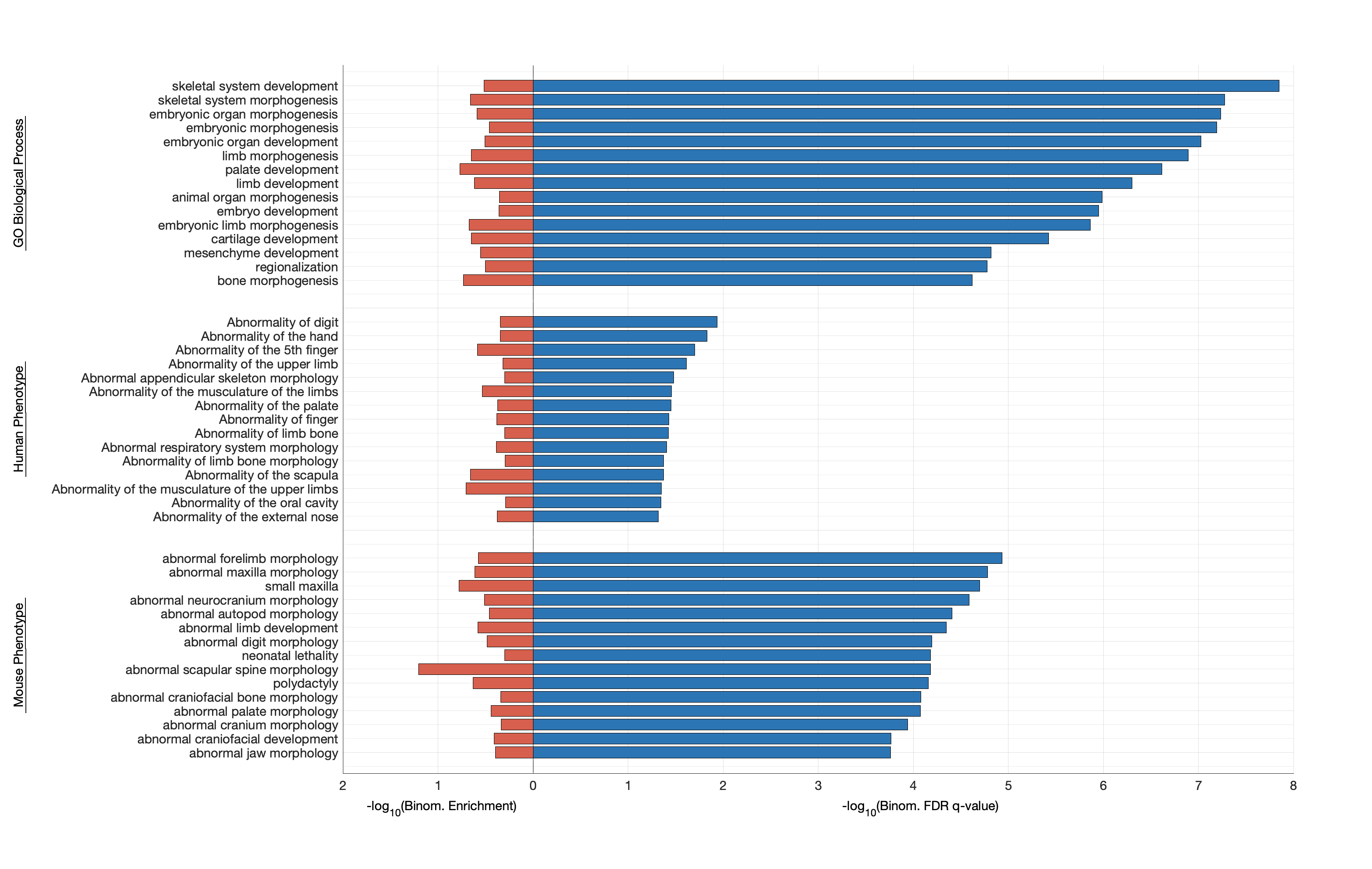

Supplement: S8 Fig — Top 15 gene ontology enrichment of biological process GO terms, human phenotypes and mouse phenotypes. Plotted is the binomial test FDR (blue) and binomial enrichment (orange). (TIF) [file pgen.1009528.s008.tif]

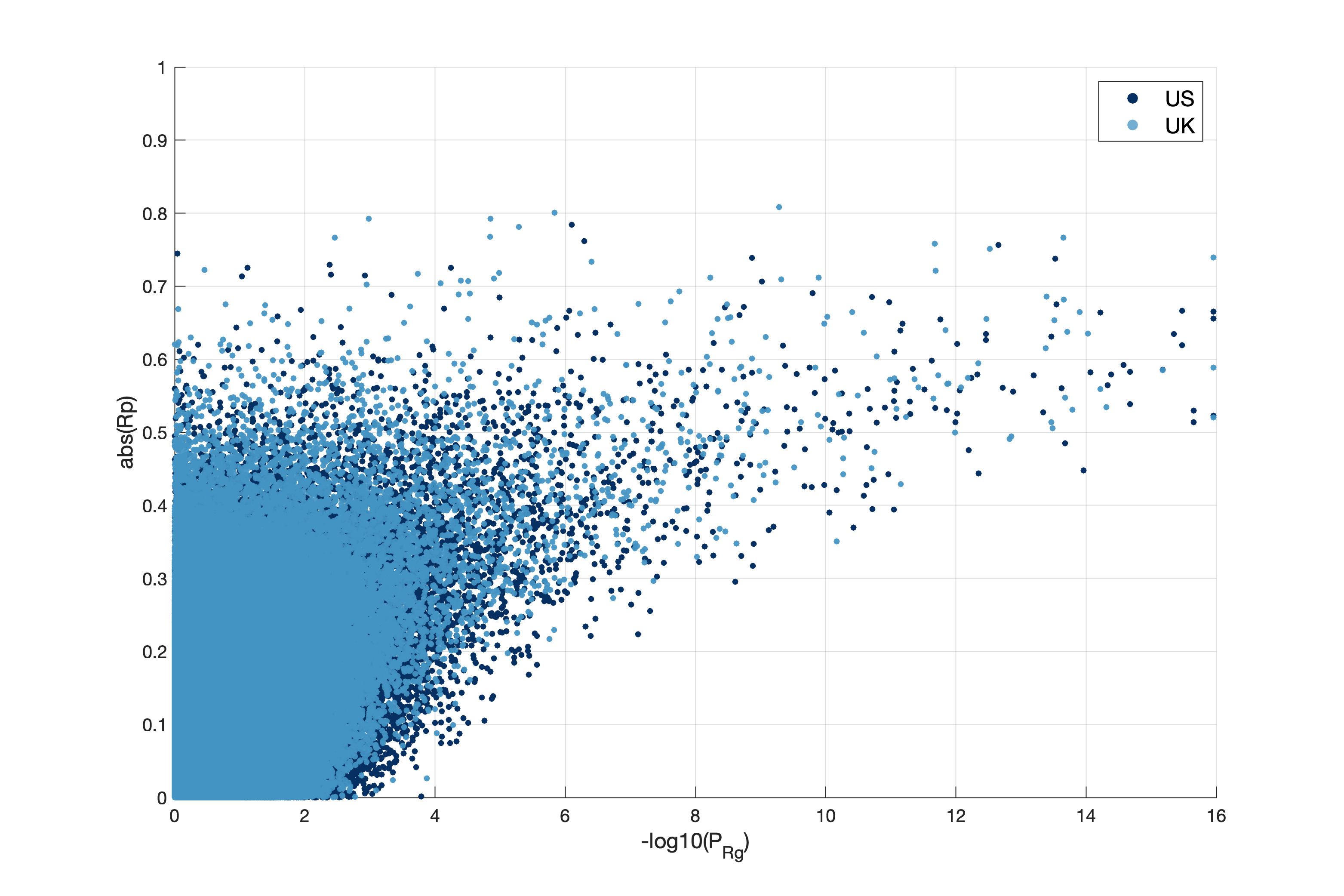

Supplement: S9 Fig — Relationship between genetic correlation p-values (x-axis) and the phenotypic correlation (y-axis) in the US and UK cohort. Pairwise correlations between traits that were derived from the same family were excluded. Rp, phenotypic correlation; Rg, genetic correlation. (TIF) [file pgen.1009528.s009.tif]

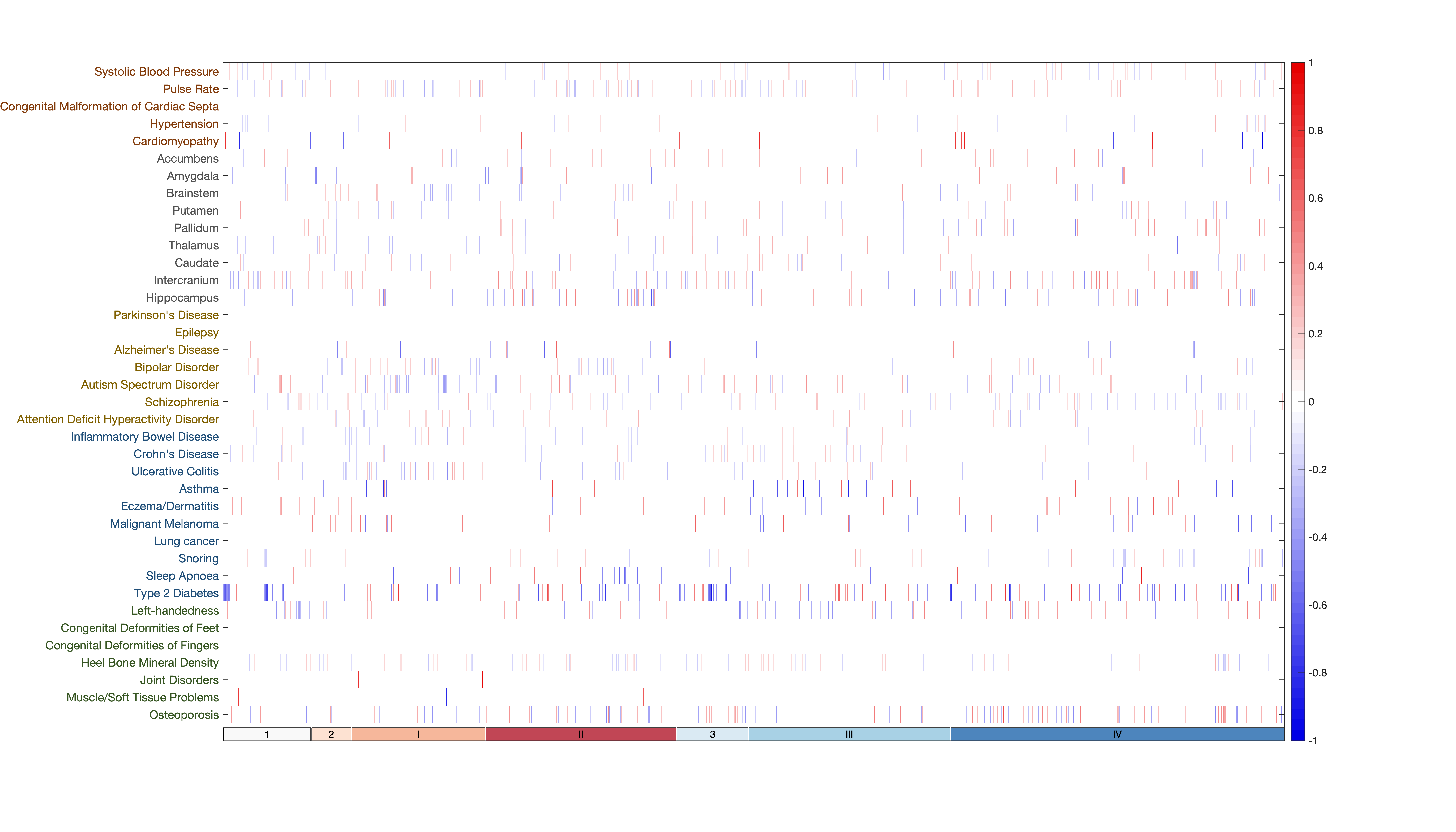

Supplement: S10 Fig — Pairwise correlations between facial and non-facial traits that reached nominal significance (p < 0.05), computed using cross-trait LDSC. Facial traits are sorted per quadrant, corresponding to the polar dendrogram displaying the facial segments in Fig 2A. (TIF) [file pgen.1009528.s010.tif]

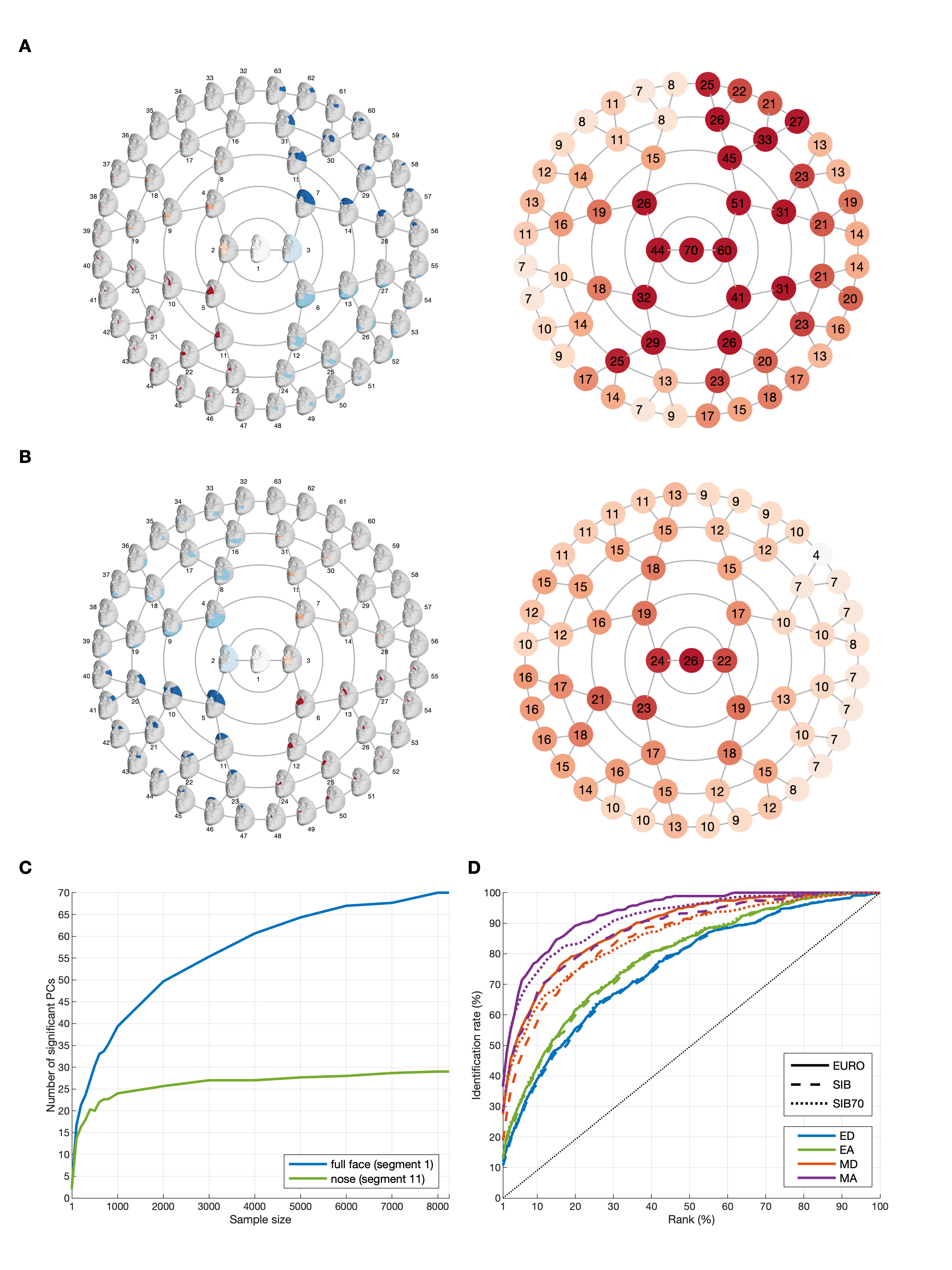

Supplement: S11 Fig — Hierarchical facial segmentation and number of significant principal components determined by parallel analysis in the (A) EURO (n = 8,246) and (B) SIB (n = 424) cohort. (C) Number of significant components retained by parallel analysis in varying, randomly generated subsets of the EURO cohort. (D) Cumulative match characteristic curves for full-face matchings (segment 1) of siblings based on the Mahalanobis angle in a EURO-based (solid line) and SIB-based (dashed line) reference space. In the ‘SIB70’ space (dotted line), the number of dimensions is equal to the original EURO reference space. ED, Euclidean distance; EA, Euclidean angle; MD, Mahalanobis distance; MA, Mahalanobis angle. (TIF) [file pgen.1009528.s011.tif]

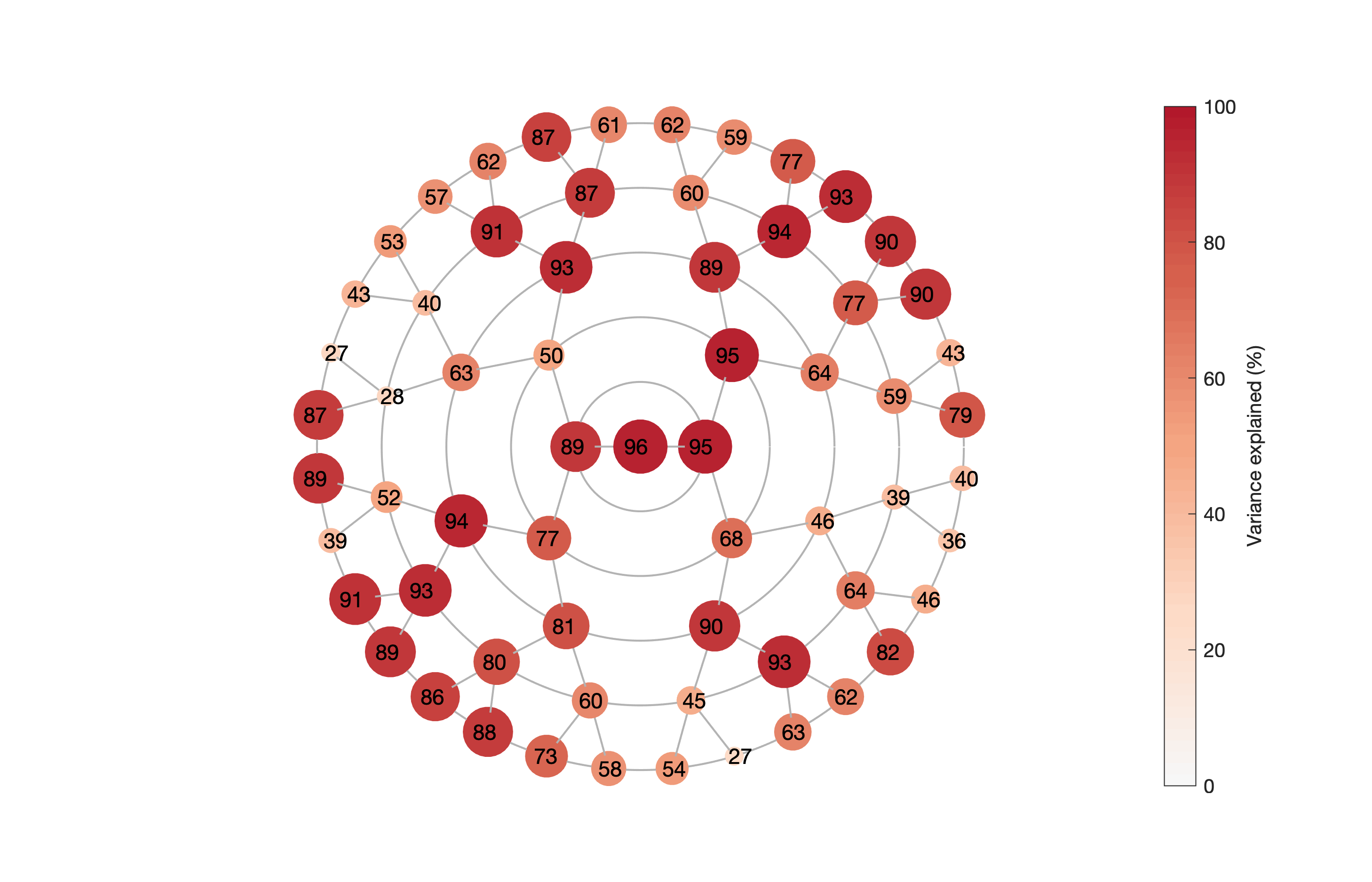

Supplement: S12 Fig — The amount of variation explained by the sib-shared traits expressed as percentage for each facial segment. The structure of the rosette plot corresponds to the polar dendrogram displaying the facial segments in Fig 2A. (TIF) [file pgen.1009528.s012.tif]

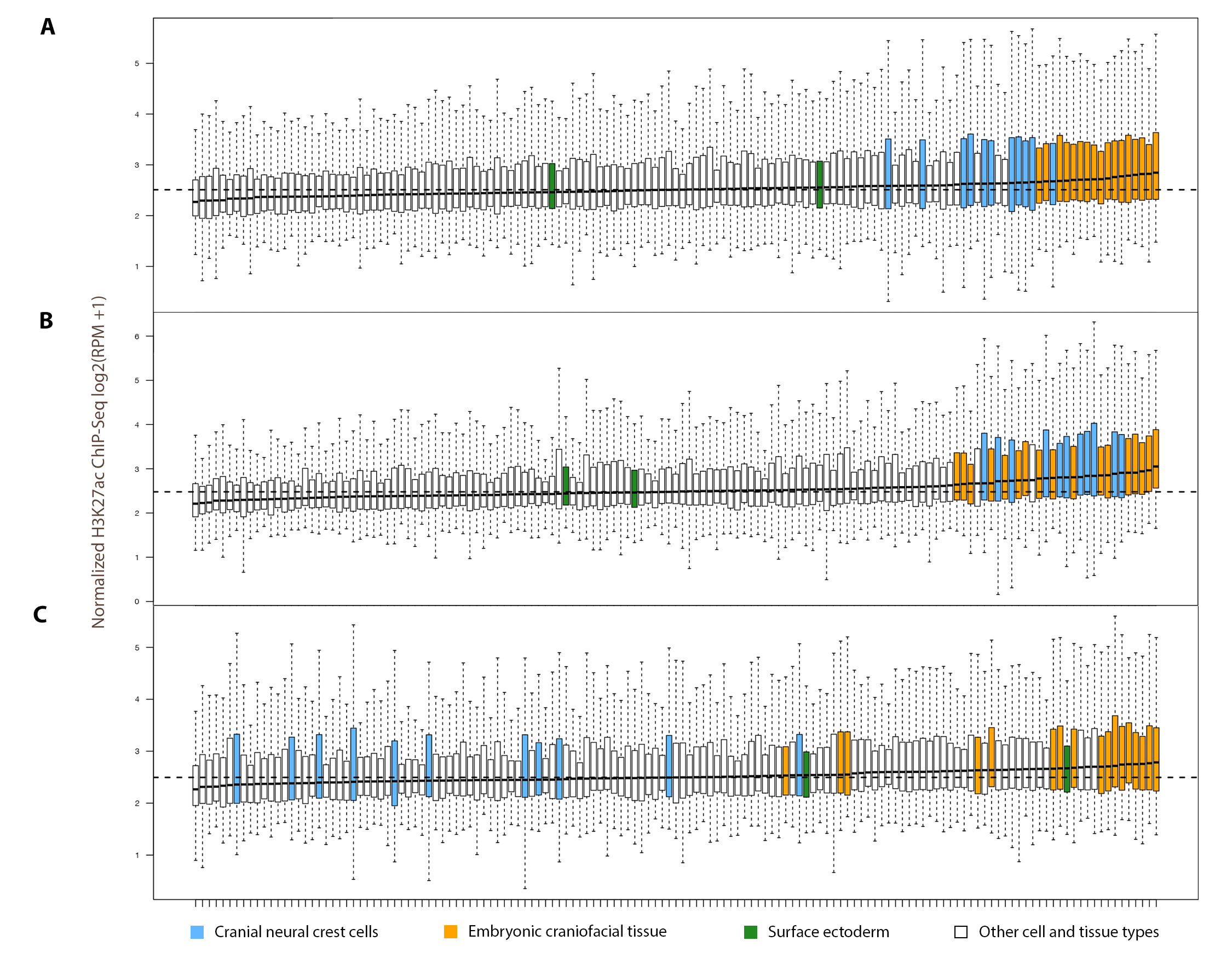

Supplement: S13 Fig — Shown are the boxplots of the distribution of H3K27ac ChIP-seq signals in 20 kb regions around the (A) 218 lead SNPs, (B) 100 overlapping SNPs and (C) 118 non-overlapping SNPs in various adult, embryonic and in vitro–derived cell types. Overlap was determined with reference to the study of White et al. [25], who utilized the same European study cohort in a multivariate GWAS. Samples corresponding to CNCCs (blue), embryonic craniofacial tissue (orange) and surface ectoderm (green) are highlighted. (TIF) [file pgen.1009528.s013.tif]

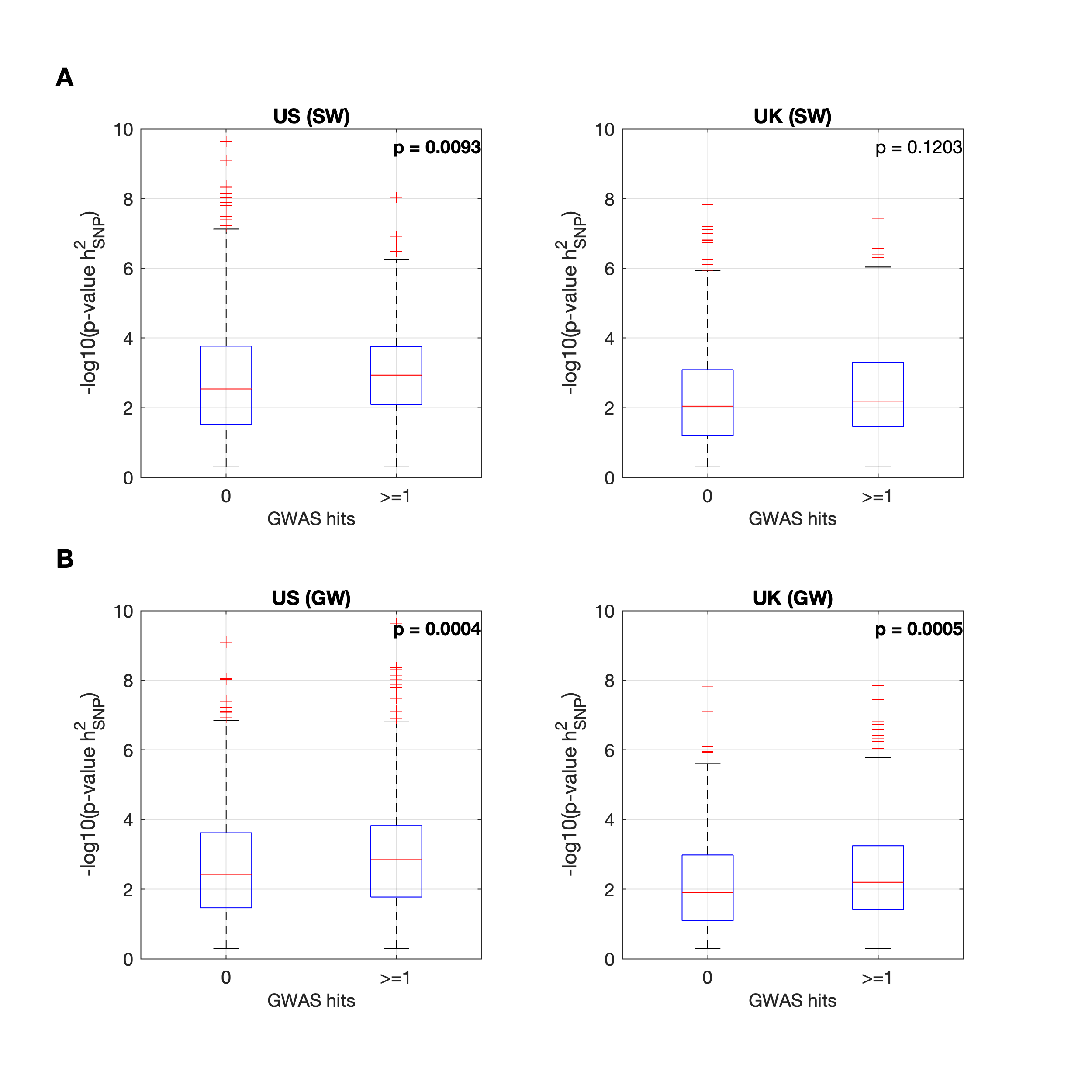

Supplement: S14 Fig — Link between SNP-based heritability and (A) study-wide (‘SW’) and (B) genome-wide (‘GW’) significance of sib-shared traits in the GWAS meta-analysis. Traits that didn’t reach statistical significance in the GWAS are coded as ‘0’; traits that were associated with at least one of the identified loci are coded as ‘> = 1’. The two-sample t-test p-value is plotted on top of each panel, with significant values indicated in bold. (TIF) [file pgen.1009528.s014.tif]
