# Supplementary material for: 3D facial phenotyping by biometric sibling matching used in contemporary genomic methodologies
Source: PLoS Genet. 2021 May 13;17(5):e1009528. doi: 10.1371/journal.pgen.1009528 (PMC8118281; doi:10.1371/journal.pgen.1009528)
Supplement: S1 Text — (PDF) [file pgen.1009528.s024.pdf]

## S1 Text. Exome-wide low frequency variant analysis.

### Supplementary Results

For the analysis of low-frequency variants, we used a gene-based testing approach in a subsample of the EURO cohort (n = 1,906) with exome-wide sequencing data available. A total of 53 genes passed the exome-wide significance threshold ($p < 3.9 \times 10^{-6}$), yet none surpassed the strict study-wide significance level adjusted for the number of independent tests ($p < 1.2 \times 10^{-8}$; S5 Table) [1]. Two genes, *GAD1* and *N4BP2*, are known craniofacial cleft candidates [2–4], and *ESR1* has been shown to be associated with facial features by several others [5,6]. We also noted that five genes identified (*ARSJ, TRIM55, TLE3, NBN* and *TMX2*), when disrupted, can cause different human syndromes involving craniofacial phenotypes. For example, *NBN* underlies the Nijmegen breakage syndrome, characterized by distinctive facial features including a prominent nose [7]. The same gene in our study was also associated with aspects of nasal shape (trait 577, segment 23).

To explore the potential connection of signals from common and rare variants, we inspected the GWAS results in the 500 kb flanking regions of these 53 genes. For five loci (near the genes *PTPN4, EHHADH, CRHR2, MLLT1* and *OR10A2*) a GWAS signal and low-frequency signal were observed in proximity, indicating that both common and rare variants may contribute to facial variation at these loci. Although none of these showed an association with the exact same sib-shared trait, some were in overlapping facial areas (S5 Table). For example, coding low-frequency variants of *MLLT1* were associated with variation in the full face (trait 48, segment 1), while the GWAS locus immediately downstream of *MLLT1* was associated with the corners of lips (trait 766, segment 39). Interestingly, this GWAS locus overlaps with potential craniofacial enhancers from in-vitro derived CNCCs, and thus may have a regulatory role on *MLLT1* [8].

No overlap was observed with previous work, in which a similar analysis of low-frequency variants in the same cohort was conducted, but using multivariate modular phenotypes [9]. This is reasonable in that, unlike common SNPs, low-frequency and rare variants are expected to have more unique effects and the traits under study were highly distinct. Similarly, the sib-shared traits were originally defined in a separate cohort (SIB), independent of those individuals being tested for genetic association (EURO). Therefore, low-frequency and rare

variants affecting the sib-shared traits may simply not be present in the genotyped sample, which may in part explain the lack of study-wide significant signals.

**Supplementary Methods**

We conducted gene-based tests of low-frequency variants across the whole exome in the PITT sample (n = 1,906). Other cohorts were not included due to a lack of exome data. Imputed genotypes with a certainty above 0.9 were used to fill in any sporadic missingness among genotype calls of the directly genotyped variants. We did not include any wholly unobserved, imputed SNPs in this analysis. SNPs with MAF $< 0.01$ were grouped into genes and genes with at least two SNPs were then tested for association with the sib-shared traits, using SKAT-O [10], an optimal unified approach combining the sequence kernel association test and burden test implemented in rvtests [11]. The analysis comprised a total of 79,661 variants in 12,698 genes. The exome-wide and study-wide significance thresholds were therefore set to be $p < 3.9 \times 10^{-6}$ (i.e. $p < 0.05/12,698$) and $p < 1.2 \times 10^{-8}$ (i.e. $p < 3.9 \times 10^{-6}$ divided by 322, corresponding to the number of independent traits), respectively.

**References**

1. Li J, Ji L. Adjusting multiple testing in multilocus analyses using the eigenvalues of a correlation matrix. Heredity. 2005;95(3):221–227.

2. Oh W-J, Westmoreland JJ, Summers R, Condie BG. Cleft palate Is caused by CNS dysfunction in Gad1 and Viaat knockout mice. Laudet V, editor. PLOS ONE. 2010;5(3):e9758.

3. Leslie EJ, Carlson JC, Shaffer JR, Buxó CJ, Castilla EE, Christensen K, et al. Association studies of low-frequency coding variants in nonsyndromic cleft lip with or without cleft palate. Am J Med Genet A. 2017;173(6):1531–1538.

4. Suzuki A, Abdallah N, Gajera M, Jun G, Jia P, Zhao Z, et al. Genes and microRNAs associated with mouse cleft palate: a systematic review and bioinformatics analysis. Mech Dev. 2018;150:21–27.

5. Chung K, Richards T, Nicot R, Vieira AR, Cruz CV, Raoul G, et al. ENPP1 and ESR1 genotypes associated with subclassifications of craniofacial asymmetry and severity of temporomandibular disorders. Am J Orthod Dentofacial Orthop. 2017;152(5):631–645.

6. Omori MA, Gerber JT, Marañón-Vásquez GA, Matsumoto MAN, Weiss SG, do Nascimento MA, et al. Possible association between craniofacial dimensions and genetic markers in ESR1 and ESR2. J Orthod. 2020;(1):65–71.

7. Varon R, Dutrannoy V, Weikert G, Tanzarella C, Antoccia A, Stöckl L, et al. Mild Nijmegen breakage syndrome phenotype due to alternative splicing. Hum Mol Genet. 2006;15(5):679–689.

8. Prescott SL, Srinivasan R, Marchetto MC, Grishina I, Narvaiza I, Selleri L, et al. Enhancer divergence and cis-regulatory evolution in the human and chimp neural crest. Cell. 2015;163(1):68–83.

9. Liu D, Alhazmi N, Hecht JT, Wehby GL, Moreno LM, Heike CL, et al. Exome-wide low-frequency genetic variants contribute to human craniofacial morphology. Genet Epidemiol. 2019.864–925.

10. Lee S, Emond MJ, Bamshad MJ, Barnes KC, Rieder MJ, Nickerson DA, et al. Optimal unified approach for rare-variant association testing with application to small-sample case-control whole-exome sequencing studies. Am J Hum Genet. 2012;91(2):224–237.

11. Zhan X, Hu Y, Li B, Abecasis GR, Liu DJ. RVTESTS: an efficient and comprehensive tool for rare variant association analysis using sequence data. Bioinformatics. 2016;32(9):1423–1426.
