# Supplementary material for: 3D facial phenotyping by biometric sibling matching used in contemporary genomic methodologies
Source: PLoS Genet. 2021 May 13;17(5):e1009528. doi: 10.1371/journal.pgen.1009528 (PMC8118281; doi:10.1371/journal.pgen.1009528)
Supplement: S2 Text — (PDF) [file pgen.1009528.s025.pdf]

S2 Text: Genetic correlation between sib-shared traits and non-facial traits and diseases.

Supplementary Results

Genetic correlations between sib-shared facial traits and non-facial traits and diseases (n = 38; S7 Table) were computed using LDSC [1,2]. Given the relatively modest sample size of the combined EURO cohort (n = 8,246), standard errors for genetic correlations were fairly large (mean = 0.2672) and no significant values could be observed after adjustment for multiple testing ($p < 4.09 \times 10^{-6}$; S10 Fig and S8 Table). However, relaxation of the significance threshold to an arbitrary threshold of $p < 1 \times 10^{-3}$ revealed some evidence for genetic correlation with interesting phenotypes (Table A). For example, handedness has previously been associated with lower face variability or bilateral retrognathism, likely mediated through tuberculosis susceptibility [3]. In the same way, we found a negative correlation between left-handedness and a protrusion of the lower face. In children, particular facial features (e.g. long face, reduced nose prominence and width, reduced mandibular prominence) have also been described in the context of sleep-disordered breathing with symptoms including snoring, mouth breathing and sleep apnea [4]. A correlation between chin morphology and snoring was identified, although the observed effect was in the opposite direction. We also noted a correlation between facial morphology, intercranial volume and autism spectrum disorder, possibly reflecting the close connection between the face and brain during embryological development [5]. Others reflected a connection to cardiovascular and bone-related disorders (e.g. osteoporosis) and traits.

Table A. Genetic correlation among facial and non-facial traits with $p < 0.001$.

Sib-shared Trait	Non-facial Trait	R_g	SE	P-value	Sib-shared Trait	Non-facial Trait	R_g	SE	P-value
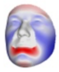 189	Left-handedness	-0.490	0.124	7.72E-05	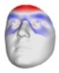 275	Pulse Rate	0.358	0.105	7.00E-04
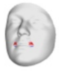 770	Pulse Rate	-0.317	0.089	3.00E-04	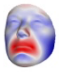 76	Left-handedness	-0.355	0.105	7.00E-04
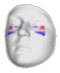 1020	Systolic Blood Pressure	-0.226	0.063	3.00E-04	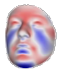 58	Autism Spectrum Disorder	0.399	0.119	8.00E-04
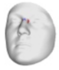 779	Osteoporosis	-0.470	0.132	4.00E-04	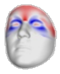 293	Osteoporosis	0.480	0.145	9.00E-04
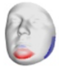 615	Snoring	0.317	0.089	4.00E-04	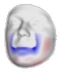 245	Intercranial Volume	-0.410	0.123	9.00E-04
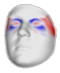 475	Osteoporosis	0.471	0.135	5.00E-04					

Sib-shared traits are visualized using colormaps representing the normal displacement in each 3D point on the facial surface. Going from the inverse face (*anti-face*) to the sibling average (*face*), red indicates an outward displacement, while blue indicates an inward displacement. The index of the corresponding sib-shared trait is shown below each facial map. R_g, genetic correlation; SE, standard error.

Supplementary Methods

Genetic correlations between sib-shared traits and other publicly available traits were computed using cross-trait LDSC (version v1.0.1) [1,2]. LD scores were readily obtained from the 1000 Genomes European data [6] and SNPs were filtered to HapMap3 SNPs [7]. While the bivariate GREML method requires individual level data, LDSC utilizes GWAS summary statistics to estimate the genetic correlation. Summary statistics of a diverse set of traits and diseases (n = 38) were downloaded, including limb-related phenotypes, connective tissue disorders, MRI-derived brain volumes, neurodevelopmental disorders, cancer types, glycemic traits, sleeping disorders, autoimmune diseases, and cardiovascular traits (S7 Table). The significance threshold correcting for multiple testing was determined at $p < 4.09 \times 10^{-6}$ (i.e. $p < 0.05/(38 \times 322)$, where 38 is the number of publicly available traits and 322 is the number of independent sib-shared facial traits).

References

1. Bulik-Sullivan BK, Loh P-R, Finucane HK, Ripke S, Yang J, Schizophrenia Working Group of the Psychiatric Genomics Consortium, et al. LD Score regression distinguishes confounding from polygenicity in genome-wide association studies. Nat Genet. 2015;47(3):291–295.

2. Bulik-Sullivan B, Finucane HK, Anttila V, Gusev A, Day FR, Loh P-R, et al. An atlas of genetic correlations across human diseases and traits. Nat Genet. 2015;47(11):1236–1241.

3. Hujoel PP. Handedness and lower face variability: Findings in three national surveys. Laterality Asymmetries Body Brain Cogn. 2018;23(1):113–128.

4. Al Ali A, Richmond S, Popat H, Playle R, Pickles T, Zhurov AI, et al. The influence of snoring, mouth breathing and apnoea on facial morphology in late childhood: a three-dimensional study. BMJ Open. 2015;5(9):e009027.

5. Marcucio R, Hallgrimsson B, Young NM. Facial Morphogenesis: Physical and molecular interactions between the brain and face. Curr Top Dev Biol. 2015;115:299–320.

6. McVean GA, Altshuler DM, Durbin RM, Abecasis GR, Bentley DR, Chakravarti A, et al. An integrated map of genetic variation from 1,092 human genomes. Nature. 2012;491(7422):56–65.

7. Altshuler DM, Gibbs RA, Peltonen L, Dermitzakis ET, Schaffner SF, Yu F, et al. Integrating common and rare genetic variation in diverse human populations. Nature. 2010;467(7311):52–58.
